# Fasting, ketogenic, and anti-inflammatory diets in multiple sclerosis: a randomized controlled trial with 18-month follow-up

**DOI:** 10.1186/s40795-025-01156-5

**Published:** 2025-08-20

**Authors:** Lina S. Bahr, Judith Bellmann-Strobl, Daniela A. Koppold, Rebekka Rust, Tanja Schmitz-Hübsch, Maja Olszewska, Jean Stadlbauer, Markus Bock, Michael Scheel, Claudia Chien, Jan Multmeier, Alexander Krannich, Andreas Michalsen, Friedemann Paul, Anja Mähler

**Affiliations:** 1 Experimental and Clinical Research Center (ECRC), Charité – Universitätsmedizin Berlin, corporate member of Freie Universität Berlin and Humboldt- Universität zu Berlin, Lindenberger Weg 80, 13125 Berlin, Germany; 2 Institute of Social Medicine, Epidemiology, and Health Economics, corporate member of Freie Universität Berlin and Humboldt-Universität, Charité - Universitätsmedizin, Berlin, Germany; 3 Max Delbrück Center for Molecular Medicine in the Helmholtz Association (MDC), Berlin, Germany; 4 Experimental and Clinical Research Center, Max Delbrück Center for Molecular Medicine in the Helmholtz Association and Charité - Universitätsmedizin Berlin, Chariteplatz 1, 10117 Berlin, Germany; 5 BioStats GmbH, Nauen, Germany; 6 Department of Internal Medicine and Nature-based Therapies, Immanuel Hospital Berlin, Berlin, Germany; 7 Department of Pediatrics, Division of Oncology and Hematology, Charité – Universitätsmedizin Berlin, Corporate Member of Freie Universität Berlin and Humboldt- Universität zu Berlin, Berlin, Germany; 8 Institut für Med. Immunologie, Immundefektambulanz, Charité – Universitätsmedizin Berlin, Campus Virchow-Klinikum (CVK), Augustenburger Platz 1, 13353 Berlin, Germany; 9 Department of Hand Surgery, Upper Extremity and Foot Surgery, Center for Orthopedic Rheumatology and Trauma Surgery, Hospital Waldfriede, Argentinische Allee 40, 14163 Berlin, Germany; 10 Department of Endocrinology and Metabolic Diseases, Charité - Universitätsmedizin Berlin, Berlin, Germany; 11 Neuroscience Clinical Research Center, Charité – Universitätsmedizin Berlin, Berlin, Germany; 12 Charité – Universitätsmedizin Berlin, corporate member of Freie Universität Berlin and Humboldt- Universität zu Berlin, Berlin, Germany

**Keywords:** Anti-inflammatory diet, Dietary intervention, Fasting, Caloric restriction, Ketogenic diet, Multiple sclerosis, NfL, Cognition, MRI, Cardiometabolic risk

## Abstract

**Background:**

Multiple sclerosis (MS) is the most common inflammatory disease of the central nervous system in young adulthood leading to disability and early retirement. Ketone-based diets improve the disease course in MS animal models and health outcomes in different pilot studies of neurodegenerative diseases.

**Methods:**

We enrolled 105 individuals with relapsing-remitting MS (RRMS) in an 18-month, randomized, controlled study, and randomized them into (1) standard healthy diet (SD) as recommended by the German Nutrition Society, (2) fasting diet (FD) with 7-day fasts every 6 months with intermittent fasting at 6 of 7 days a week or (3) ketogenic diet (KD) with 20–40 g carbohydrates per day. Primary outcome was the number of new MRI lesions after 18 months in the KD and FD compared to SD and compared to baseline. Secondary outcomes included further MRI outcomes, disease biomarkers as well as metabolic, and clinical MS outcomes.

**Results:**

Eighty-one participants completed the study. The primary endpoint number of new T2 lesions after 18 months did not change in any of the groups (SD 0 (0-(-1)), FD 0 (2 − 0), KD 0 (2 − 0)). Secondary endpoints were analyzed exploratorily: Compared to baseline, in the FD group, Neurofilament light chain (NfL) -concentrations were lower at 9 months (-1.94 pg/mL, *p* = 0.042) and depressive symptoms improved slightly at 18 months (*p* = 0.079). In the KD group, cognition improved at 18 months (symbol digit modalities test + 3.7, *p* = 0.020). Cardiometabolic risk markers (body mass index, abdominal fat, blood lipids, adipokines, blood pressure) improved in all three groups at 9 months differently and were partially associated with clinical outcomes in the FD and KD group.

**Conclusion:**

The results suggest beneficial effects of dietary interventions, underscoring their potential as a complementary strategy in the treatment of RRMS. To further clarify the impact of such interventions on the disease course and patient-centered outcomes — such as cognitive function and depressive symptoms —future studies with larger, more homogeneous study populations are warranted.

**Trial registration:**

ClinicalTrials.gov, NCT03508414. Retrospectively registered on 25 April 2018.

**Supplementary Information:**

The online version contains supplementary material available at 10.1186/s40795-025-01156-5.

## Introduction

Multiple sclerosis (MS) is an autoimmune inflammatory and neurodegenerative disorder of the central nervous system caused by a combination of genetic susceptibility and environmental factors [1]. Many individuals with MS are highly motivated to incorporate complementary approaches, such as specific diets, into their treatment. However, despite 70 years of research efforts in this field, there is still no evidence-based diet showing a beneficial effect on disease progression. Current dietary recommendations, such as the Mediterranean diet, aim at decreasing the risk of comorbidities, and thereby secondarily ameliorate prognosis [2]. However, robust evidence of effects of healthy, anti-inflammatory diets on MS disease pathology itself is still lacking [3, 4].

In contrast, emerging data on fasting diets (FD) and ketogenic diets (KD) indicate direct effects. Both FD and KD drastically reduce carbohydrate intake, initiating ketone body production from internal and/or external fats, the state of ketosis. These ketone bodies may provide an alternative, more efficient, energy source for the brain, which might be important for the regeneration of demyelinated axons [5].

First indicators on the effectiveness of FDs in MS come from animal experimental studies. A calorie restriction of 40% ameliorated the clinical disease course of murine experimental autoimmune encephalomyelitis (EAE). Calorie restricted mice showed less severe inflammation, demyelination, and axonal injury [6]. In addition, alternate day fasting in EAE mice reduced incidence, onset, and severity of the disease [7]. A similar study confirmed these results and suggested that intermittent fasting should be initiated shortly after disease onset [8].

Fitzgerald et al. investigated safety, feasibility, weight loss and participant reported outcomes after 8 weeks of calorie restriction and intermittent fasting in individuals with MS. Both diets caused significant weight loss and increased emotional well-being [9].

KDs seem to be equally promising. KD was established as an alternative treatment for pharmaco-resistant childhood epilepsy in the 1920s [10]. This was based on the observation that seizures stopped during fasting, but extended fasting periods in children were obviously not reasonable. Consequently, KD was developed to mimic biochemical effects caused by fasting [11]. KDs also seem to improve symptoms of neurodegenerative diseases, such as Alzheimer´s and Parkinson´s disease [12, 13]. EAE mice on KD showed ameliorated disease progression, motor disability, hippocampal atrophy, lesion load, inflammation, and oxidative stress [14]. In our own study we investigated feasibility, safety and health-related quality of life associated with a 6-month FD and KD intervention in individuals with relapsing-remitting MS (RRMS) [15]. A single-arm, uncontrolled study reported improved metabolic and neurological outcomes after 6 months of a modified Atkins diet in individuals with RRMS [16, 17].

Based on this evidence, we designed a large-scale, long-term, randomized, controlled clinical study to investigate the effects of a FD and a KD compared to a standard healthy diet (SD) with anti-inflammatory focus in individuals with RRMS. We hypothesized that both FD and KD are superior to SD with respect to new cerebral lesions on cranial T2-weighted magnetic resonance imaging (MRI) after 18 months study interventions compared to baseline [18].

## Materials and methods

### Study design and published work

The Nutritional Approaches in MS (NAMS) study was a single center, randomized, controlled, parallel group study (ClinicalTrials.gov, NCT03508414). Participants were randomized to either SD, FD, or KD. The study was conducted at the Neuroscience Clinical Research Center of Charité – Universitätsmedizin Berlin from April 2017 to October 2021 (last visit). Beside the study protocol [18], an interim analysis on the effects of the dietary interventions on neuropsychiatric symptoms was published in the scope of a doctoral thesis [19]. The study was conducted in part during the COVID-19 pandemic. To ensure transparent reporting of trial modifications, the CONSERVE extension of the CONSORT guidelines was applied. A completed CONSERVE checklist is provided in the supplement.

In response to government-imposed travel restrictions, the study team, under the direction of the principal investigator, implemented protocol adjustments: between March 2020 and December 2021, dietary group sessions were temporarily conducted in a virtual format. To mitigate potential impacts on adherence, several measures were implemented. These, included enhancing opportunities for peer interaction during virtual group sessions and improving the accessibility of the study team -including dietary counselors and physicians – through increased availability via phone and online consultations.

### Participants

Main inclusion criteria were (1) definite diagnosis of RRMS according to the 2017 revised McDonald criteria [20] and (2) stable disease modifying therapy (DMT) or no DMT for at least six months prior to enrolment, (3) recent disease activity had to be confirmed, defined as at least one new lesion on cranial MRI or at least one clinical relapse within the last two years prior to enrolment, (4) Expanded Disability Status Scale score (EDSS) had to be < 4.5. Key exclusion criteria were start or change of DMT within six months before study start or during the study. The complete inclusion and exclusion criteria are listed in the published protocol [18]. The study was approved by the institutional review board of Charité – Universitätsmedizin Berlin (EA1/200/16). All research was performed in accordance with relevant guidelines/regulations and in accordance with the Declaration of Helsinki. Informed consent was obtained from all participants after screening to take part in the study and that pseudonymized data can be published. Recruitment efforts involved active outreach through medical practices and media engagement; however, these activities were partially limited by travel restrictions imposed during the pandemic.

### Randomization

Participants were stratified according to DMT use (yes or no), sex (male or female), and T2 lesion load (low (≤ 15) vs. high (> 15)), to equally distribute potential confounder. Block randomization in treatment groups (1:1:1) was done by an external statistician, who was not involved in any study procedures. Whereas participants and study personnel could obviously not be blinded to the dietary intervention, outcome assessors (MRI, EDSS) were trained and blinded.

### Dietary interventions

Dietary interventions were instructed in 10 group sessions over 18 months. The group setting was chosen to encourage study adherence, explain dietary interventions, provide support, assess adverse events, and validly evaluate compliance. From March 2020 to December 2021, dietary groups sessions were conducted virtually, due to government-imposed travel restrictions. A detailed description of the interventions can be found in the study protocol [18].

The SD group followed a healthy, mainly vegetarian-focused diet according to the recommendations of the German Society for Nutrition (DGE). Participants were additionally instructed to adapt their omega-6 to omega-3 fatty acid intake to reach a ratio of 5:1 to set an anti-inflammatory focus. Total energy intake was not restricted. Following intermittent fasting patterns and/or any other diets was prohibited, thereby ensuring discrimination between interventions. The SD group, functionally interpreted as our control group, received an intervention for recruitment and ethical reasons (an 18-months follow-up of participants not receiving any intervention would have been unethical and hardly feasible).

The FD group fasted every 6 months for 7 days according to Buchinger [21]. Participants in this group attended additional group sessions every second day during the 7-day fasts. Between these fasts, participants practiced 14:10 time-restricted eating on 6 days each week. Dietary intake recommendations were the same as in the SD group.

The KD group reduced their carbohydrate intake to 20–40 g/d and increased their fat intake to 70–80% of total energy intake. A focus on plant-based fats was advised. Participants were allowed to gradually increase their carbohydrate intake up to their individual limit (mostly at 40 g/d, maximum 50 g/d) to maintain stable ketosis, defined as ≥ 0.5 mmol/L blood beta-hydroxybutyrate (BHB). For self-monitoring, participants received a standard hand-held ketone-meter (GlucoMen LX plus). Ketone values were discussed and followed-up during study visits and dietary group sessions by the study team. The state of ketosis and measuring of blood ketones allowed to evaluate compliance according to a biomarker.

### Outcome parameters and assessment methods

The primary endpoint and all secondary endpoints were measured at baseline, after 9 and 18 months. A detailed list of all study assessments with assessment time points can be found in the study protocol SPIRIT flow diagram [18].

#### Primary outcome

Primary outcome was the number of new T2-hyperintense lesions found in cranial MRI after 18 months on FD or KD compared to SD.

#### Secondary outcomes

#### MRI outcomes and neurodegeneration markers

Brain atrophy was determined by percentage of brain volume change (PBVC) at 18 months vs. baseline. To increase internal validity of the primary endpoint, the secondary MRI endpoint change of lesion volume was amended during the study. Enlarged and/or aggregated lesions may result in unchanged or even reduced lesion counts but signify disease progression. By controlling for lesion volume, data analysis was adjusted for this potential systematic error. All MRI scans were done in a 3-Tesla MRI scanner (Tim Trio, Siemens, Erlangen, Germany) and lesions were segmented by two experienced and blinded MRI technicians.

Serum neurofilament light chain (sNfL) was assessed retrospectively after completion of the study in the accredited laboratory (Labor Berlin, Charité Vivantes GmbH and Charité – Universitätsmedizin Berlin) according to standard procedures (Quanterix NFLight^®^ Assay).

#### Details on MRI acquisition and processing

Cerebral MRI scans were performed on two 3-Tesla (Siemens MAGNETOM Trio Tim and Prisma, Erlangen, Germany) scanner models, with 32-channel head coils. The Trio MRI (Scanner 1) protocol included: [1] a T1-weighted 3D magnetization prepared rapid gradient echo (MPRAGE) cerebral MRI (1 mm isotropic resolution, repetition time (TR) = 1900ms, time to echo (TE) = 3.03ms, inversion time (TI) = 900ms); [2] a 3D T2-weighted fluid-attenuated inversion recovery sequence (FLAIR) (1 mm isotropic resolution, TR = 6000ms, TE = 388ms, TI = 1800ms). The Prisma MRI (Scanner 2) protocol included: [1] a 3D MPRAGE (1 mm isotropic resolution, TR = 1900ms, TE = 3.03ms, TI = 900ms), including the upper cervical cord; [2] a 3D FLAIR (1 mm isotropic resolution, TR = 6000ms, TE = 390ms, TI = 2100ms). For each participant at each timepoint, FLAIR images were co-registered with MPRAGE images, in which each follow-up MPRAGE was co-registered to the baseline MPRAGE in Montreal Neurological Institute (MNI) space using FMRIB’s Linear Image Registration Tool (FSL version 5.0.9). Whole brain T2-weighted hyperintense lesion masks were manually segmented from co-registered FLAIR scans using ITK-SNAP (www.itksnap.org) by 2 MRI technicians (over 10 years of MS research experience). Lesion counts and volumes extraction were performed on binary lesion masks using cluster and fslstats tools (https://fsl.fmrib.ox.ac.uk/fsl/fslwiki/Fslutils).

#### Participant -centered outcomes

##### Functional and clinical outcomes

Hand grip strength was assessed with a standard hand dynamometer (Jamar smart hand [22]) and walking endurance with the 6-min walk test (6MWT [23]), . Neurological–functional disability was assessed using the EDSS and the Multiple Sclerosis Functional Composite (MSFC [24]). The MSFC evaluates multiples domains of functional impairment in individuals with MS, including gait speed (Timed 25-Foot Walk, T25FT), fine motor skills (9-Hole Peg Test, 9HPT) and cognitive function (Paced Auditory Serial Addition test, PASAT). The MSFC score was calculated based on z-scores and referenced against baseline values from the entire study population. Cognition (cognitive processing speed) was assessed with the oral Symbol Digit Modalities Test (SDMT [25]), .

Clinical relapses were assessed by the study physicians according to established criteria.

##### Participant -reported outcomes

The presence of depressive symptoms was assessed with the Beck Depression Inventory-II (BDI-II [26]), , which is interpreted according to the following ranges: 0–8 (no depressive symptoms); 9–13 (minimal depressive symptoms); 14–19 (mild depressive symptoms); 20–28 (moderate depressive symptoms); 29–63 (severe depressive symptoms) [27].

Fatigue was assessed using the fatigue severity scale (FSS), with scores ≥ 4 indicating moderate-to-severe fatigue [28].

Mental and physical health related quality of life were assessed with the MSQol-54, an established tool to assess MS-related quality of life. It includes 54 items that can be summed up to 12 multi-item scales (physical health, physical role limitations, emotional role limitations, pain, emotional well-being, energy, social function, cognitive function, health perception, health distress, overall quality of life, sexual function) and two single items (change in health, sexual function satisfaction). Two summary scores – physical health composite and mental health composite – can be derived from a weighted combination of scale scores [29]. MSQoL-54 scale scores were created using the Likert method by averaging items within the scales, and then row scores were linearly transformed into 0-100 scales. Higher values indicate better quality of life.

#### Cardiometabolic health, anthropometry and body composition

Blood glucose, BHB, insulin, adiponectin, leptin, total cholesterol, LDL cholesterol, HDL cholesterol and triglycerides were measured after a 12-h overnight fast in the accredited laboratory (Labor Berlin, Charité Vivantes GmbH and Charité – Universitätsmedizin Berlin) according to standard procedures. Metabolic markers were assessed in order to investigate their potential of mediating effects of dietary interventions. Potential mediators of dietary effects were assessed using the following parameters: body weight was measured with a standard scale (seca GmbH and Co.KG) and Body Mass Index (BMI) was calculated as weight divided by height squared (kg/m^2^). Blood pressure was recorded using automated oscillometric sphygmomanometry after a 5-minute seated rest. Body composition was evaluated via bioelectrical impedance analysis (Biacorpus RX 4000, MEDI CAL healthcare GmbH, Karlsruhe, Germany).

#### Dietary intake

Macro- and micronutrient intake was calculated from food records, in which participants recorded the amounts of all consumed foods and drinks for four consecutive days (three week days and one weekend day), using the software OptiDiet PLUS 6.1 (GOE mbH, Linden, Germany) based on the German Nutrient Database.

#### Safety of the diets and compliance

Safety was assessed by occurrence of (serious) adverse events, vital signs (blood pressure, heart rate), monitoring of body weight and markers for kidney (creatinine, uric acid, urea) and liver function (GPT, GOT, gamma-GT). Compliance for all participants was defined as attendance of at least 7 out of 10 group sessions over the study period. Food records at 0, 9 and 18 months allowed assessment of compliance with the different dietary recommendations. Compliance to the individual interventions was additionally defined as follows: SD, adherence to DGE recommendations; FD, attendance of dietary sessions during 7-day fasts; KD, 75% of study center assessed BHB concentrations ≥ 0.5 mmol/L.

### Data capture

Study data were collected and managed using REDCap electronic data capture tools hosted at Charité Berlin [30, 31].

### Samples size calculation and data analysis

Sample size calculation and data management procedures are published in the protocol publication [18]. The primary endpoint was the number of new MRI lesions at 18 months assessed in the KD and FD group each in comparison to the SD group. Analysis of the primary endpoint was based on robust linear regression analysis in the ITT population, adjusted for baseline lesion load and lesion volume (to account for enlarged and/or aggregated lesions may result in unchanged or even reduced lesion counts but signify disease progression). In contrast to the initially planned ANCOVA analysis [18], the robust regression was preferred due to non-normally distributed data. Sensitivity analysis of the primary outcome was done applying robust linear regression in the PP population. Secondary endpoint analysis was based on linear regressions and Wilcoxon signed rank tests in the FAS population. Predictor analysis was performed post hoc. We have decided not to impute for missing values in the outcome parameters. All available cases were considered. Analysis was conducted with the software R version 4.3.1 (R Core Team (2023). R: A language and environment for statistical computing. R Foundation for Statistical Computing, Vienna, Austria. URL https://www.R-project.org/) [32] and IBM SPSS statistics version 26.

## Results

**Fig. 1:**
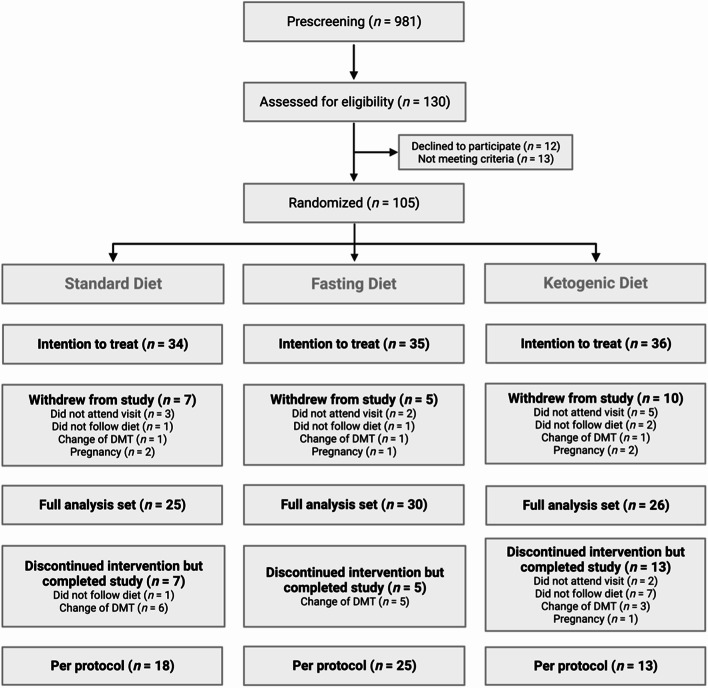
CONSORT diagram of the NAMS study. In the SD group: two MRIs were not done, which is why two more cases had to be excluded from the full analysis set. DMT, disease modifying therapy

During the recruitment period, 981 potential participants contacted the study center, of which 130 participants suited for screening and 105 were included (Fig. 1).

Baseline characteristics are displayed in Table 1. The a priori sample size of 111 participants was not reached, as the dropout rate was higher than the initially estimated 10%. Dropout rates were 21% in the SD, 14% in the FD, 28% in the KD group (Fig. 1).

There were no relevant differences in age and sex between the groups. Disease duration of the SD group was shorter than of the KD and FD group. However, EDSS score, lesion load and percentage of participants on DMT was comparable between groups. The SD group showed lower cognition (SDMT) scores (age-adjusted cut-off ≤ 55 implies cognitive impairment) and higher fatigues (FSS) scores (cut-off ≥ 4 implies moderate fatigue), compared to KD and FD group.

**Table 1:** Baseline characteristics of the NAMS intention-to-treat population

		Total	SD	FD	KD
Number of participants (n)		105	34	35	36
Age (years)	Mean (StD)	42 (9)	43 (10)	41 (9)	41 (10)
Ethnicity (Caucasian)	n (%)	100 (95)	34 (100)	34 (97)	32 (89)
Gender (female)	n (%)	88 (84)	29 (85)	28 (80)	31 (86)
Disease duration since manifestation (years)	Median (IQR)	8.0 (13.8–2.4)	5.0 (13.0-2.1)	8.9 (13.6–3.8)	8.6 (16.1-3.0)
Disease duration since diagnosis (years)	Median (IQR)	3.6 (10.3–1.3)	2.1 (5.4–1.2)	5.3 (11.1–2.1)	3.8 (10.6–1.3)
EDSS score	Median (IQR)	2.0 (2.5–1.5)	2.0 (2.5–1.5)	2.0 (2.1–1.5)	2.0 (3.1–1.5)
Number of T2-lesions	Median (IQR)	35 (75 − 19)	36 (76 − 21)	39 (67 − 23)	31 (76 − 17)
Participants with disease-modifying therapy	n (%)	60 (57)	20 (59)	19 (55)	21 (58)
*Alemtuzumab*	n (%)	1 (1)	1 (3)	0	0
*Cladribine*		1 (1)	0	1 (3)	0
*Daclizumab*		1 (1)	1 (3)	0	0
*Dimethyl fumarate*		14 (13)	2 (6)	5 (14)	7 (19)
*Fingolimod*		10 (10)	2 (6)	5 (14)	3 (8)
*Glatiramer acetate*		15 (14)	8 (24)	3 (9)	4 (11)
*Interferon beta-1a*		7 (6)	0	3 (9)	4 (11)
*Interferon beta-1b*		3 (3)	1 (3)	0	2 (6)
*Natalizumab*		4 (4)	2 (6)	2 (6)	0
*Ocrelizumab*		1 (1)	1 (3)	0	0
*Rituximab*		2 (2)	1 (3)	0	1 (3)
*Teriflunomide*		1 (1)	1 (3)	0	0
Participants without disease-modifying therapy	n (%)	45 (43)	14 (41)	16 (46)	15 (42)
Body Mass Index (kg/m^2^)	Median (IQR)	24.3 (26.6–22.2)	25.1 (26.8–22.8)	23.6 (26.3–21.6)	23.7 (26.3–22.3)
25-OH-Vitamin D3 (nmol/L)	Mean (StD)	101.0 (37.1)	105.1 (37.4)	93.9 (34.1)	104.2 (39.7)
Fatigue severity scale score	Median (IQR)	3.7 (5.3–2.2)	4.3 (5.6–1.8)	3.8 (5.2–2.8)	3.4 (4.4–2.2)
Beck depression inventory II score	Median (IQR)	7 (12 − 3)	7 (12 − 2)	9 (13 − 5)	6 (11 − 3)
Symbol digit modalities test score	Median (IQR)	56 (63 − 49)	54 (62 − 50)	57 (62 − 50)	57 (65 − 47)

EDSS, expanded disability status scale; FD, fasting diet; IQR, interquartile range; KD, ketogenic diet; SD, standard diet; StD, standard deviation, percentages of single DMTs have been rounded

### Primary outcome

Primary outcome analysis was applied in the ITT population. Treatment effects have been estimated from a robust linear regression with the number of new lesions as response, treatment group as fixed effect and adjusted for baseline number of T2-lesions and lesion volume. Treatment effects were estimated from Generalized Estimation Equations which show no difference between the groups regarding new number of T2 lesions at 18 months. Lesion volume influenced the number of new lesions at 18 months (Table 2).

**Table 2:** Robust linear regression for number of new lesions at 18 months (ITT population)

Variable	Unstandardized B	Std. Error	z value	*p*-value
**Primary analysis (ITT)**				
KD (SD as reference)	0.305	1.205	0.253	0.801
FD (SD as reference)	0.809	1.171	0.691	0.492
Lesion volume change (mm^3^)	-1.262	0.550	-2.297	0.024
Baseline lesion load (n)	-0.003	0.011	-0.259	0.797

### Sensitivity analysis

**Fig. 2:**
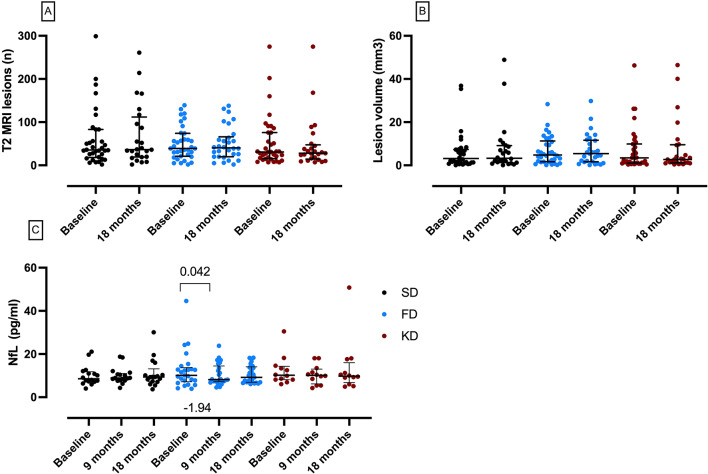
Imaging, and neurodegeneration markers in the standard diet (SD, black circles), fasting diet (FD, blue circles) and ketogenic diet (KD, red circles) of the NAMS study. (**A**) No change of the number of new MRI lesions (primary endpoint, ITT population). (**B**) No change of MRI lesion volume (ITT population). (**C**) Lower NfL concentrations at 9 months in the FD group (FAS population). **A-B**, data as median and interquartile range, *p* values by Wilcoxon signed-rank test. **C**, data as mean and standard deviation, *p* values by t-test

Sensitivity analysis investigated the per-protocol (PP) population and only emphasized significant predictive value for baseline lesion load (Table 3). The number of new T2 lesions after 18 months vs. baseline did not change in any of the groups (primary endpoint; SD 0 (0-(-1)), FD 0 (2 − 0), KD 0 (2 − 0)) (Fig. 2A). In line with this, lesion volume remained stable over 18 months in all three groups (Fig. 2B).

**Table 3:** Robust linear regression for number of new lesions at 18 months (PP population)

Sensitivity analysis (PP)	Unstandardized B	Std. Error	z value	*p*-value
KD (SD as reference)	0.055	0.344	0.160	0.873
FD (SD as reference)	0.065	0.298	0.217	0.829
Lesion volume change (mm^3^)	-0.014	0.222	-0.065	0.948
Baseline lesion load (n)	-0.011	0.003	-3.486	0.001

FD, fasting diet; ITT, intention-to-treat; KD, ketogenic diet; SD, standard diet; PP, per protocol

In addition, while the use of DMT versus no use of DMT had no significant influence, female sex and changes of lesion volume were significant predictors of the number of new lesions at 18 months in the FAS population (data not shown). No influence of the dietary interventions was observed.

### Secondary outcomes

All secondary outcomes were analyzed in the FAS population unless explicitly mentioned otherwise.

#### MRI outcomes and neurodegeneration markers

Percentage of brain volume change did not differ between the groups after 18 months study interventions (data not shown).

Serum neurofilament light chain (NfL) decreased by -1.94 ((-0.80)-0.01) pg/mL at 9 months in the FD group (Fig. 2C) but not 18 months compared to baseline. NfL concentrations at 18 months correlated positively with EDSS score (Spearman correlation, *r* = 0.370; *p* = 0.006) and MRI lesion count (Spearman correlation, *r* = 0.394; *p* = 0.003) in a pooled analysis of all groups.

#### Participant-centered outcomes

##### Functional and clinical outcomes

**Fig. 3:**
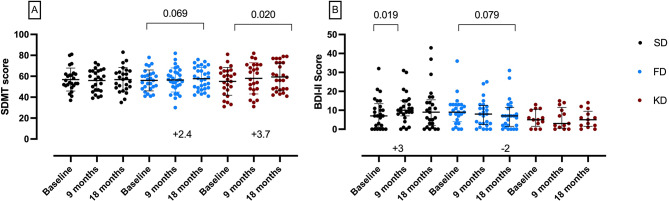
Patient-centered neuropsychiatric outcomes in the standard diet (SD, black circles), fasting diet (FD, blue circles) and ketogenic diet (KD, red circles) of the NAMS study (FAS population). (**A**) Improved cognition according to Symbol Digit Modalities Test (SDMT) at 18 months in the KD group. Data as mean and standard deviation, *p* value by t-test. (**B**) Slightly improved depressive symptoms according to Beck Depression Inventory (BDI-II) in the FD group. Data as median and interquartile range, *p* values by Wilcoxon signed-rank test

Disability status assessed by hand grip strength, 6MWT, EDSS, MSFC remained stable over 18 months across all groups. SDMT scores remained stable in the SD group, showed a slight increase in the FD group, and increased in the KD group at 18 months (Fig. 3A).

Participants reported 29 clinical relapses during the study (SD, 7 (28%); FD, 9 (30%); KD, 13 (50%)). Differences were not significant according to Chi-square tests (KD vs. FD vs. SD, *p* = 0.184; KD vs. SD, *p* = 0.186; FD vs. SD, *p* = 1; KD vs. FD, *p* = 0.210).

##### Participant-reported outcomes

BDI-II scores were slightly lower at 9 months (Unstandardized B = − 3.7, *p* = 0.065), and lower at 18 months (Unstandardized B = − 4.3, *p* = 0.015) in the FD vs. the SD group and improved over time in the FD group (Fig. 3B). FSS scores remained stable over 18 months in all groups (Suppl. Tables 3–5). There was no relevant change in mental health and physical health related quality of life (Suppl. Tables 3–5).

#### Cardiometabolic health, anthropometry and body composition

**Fig. 4:**
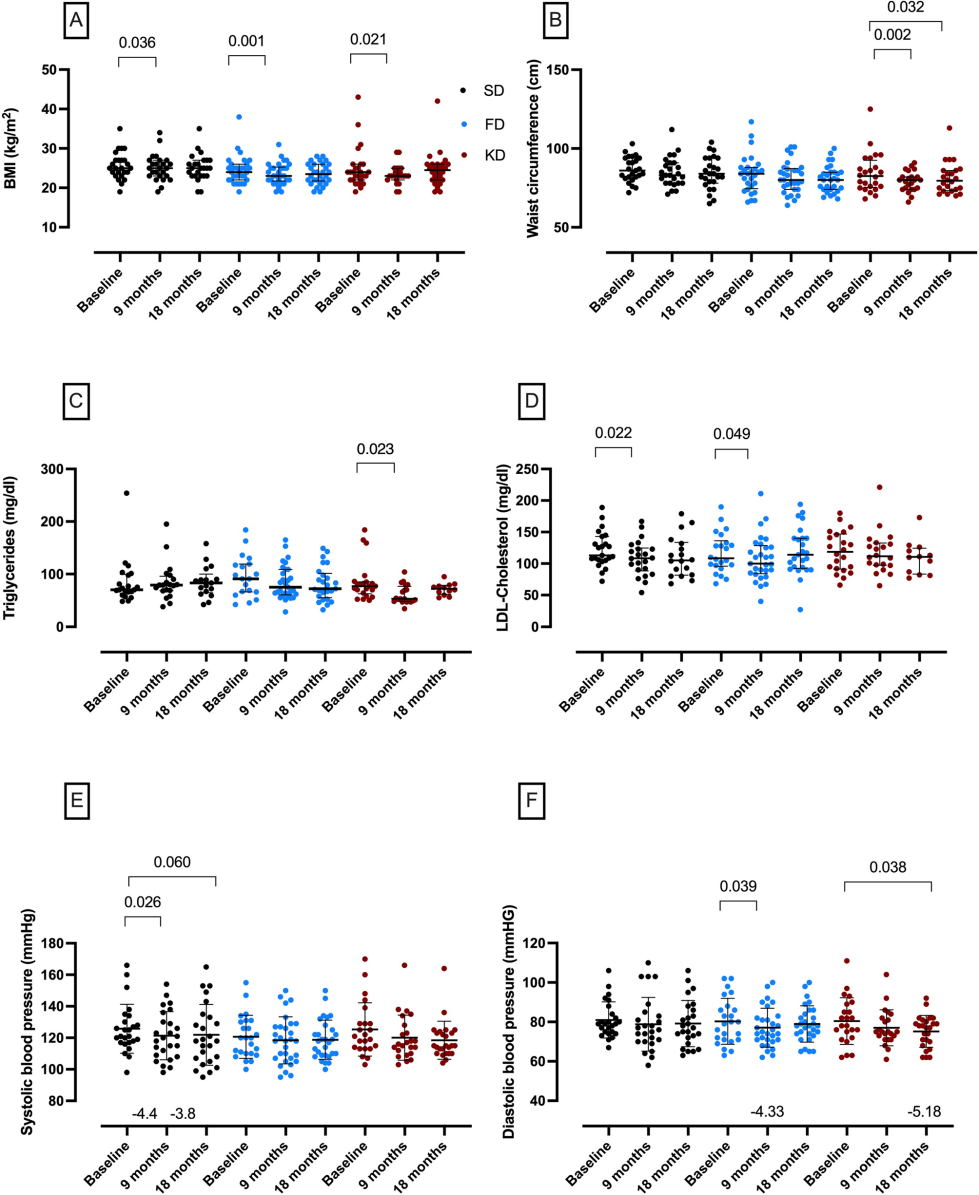
Improved cardiometabolic risk markers in the standard diet (SD, black circles), fasting diet (FD, blue circles) and ketogenic diet (KD, red circles) of the NAMS study (FAS population). (**A**) Body mass index (BMI), (**B**) waist circumference, (**C**) triglycerides and (**D**) LDL cholesterol. Data as median and interquartile range, *p* values by Wilcoxon signed-rank tests. (**E**) Systolic and (**F**) diastolic blood pressure. Data as mean and standard deviation, *p* values by t-test

Changes of cardiometabolic risk markers were most prominent at 9 months. In the KD group, triglyceride concentrations were 14 [21] mg/dL lower at 9 months vs. baseline (Fig. 4C). In the SD group, total cholesterol decreased by 12 [19] mg/dL (*p* = 0.017; Suppl. Tables 3–5)) and LDL cholesterol by 8 [14] mg/dL (*p* = 0.022) at 9 months vs. baseline (Fig. 4D). In the FD group, LDL cholesterol decreased by a median of 8.5 (7.8–11.8) mg/dL (*p* = 0.049) comparing 9 months vs. baseline (Fig. 4D). Leptin concentrations were lower (Unstandardized B = − 3.0, *p* = 0.017) and adiponectin concentrations were higher (Unstandardized B = 2.2, *p* = 0.039) in the KD vs. the SD group at 9 months, but not at 18 months. Leptin decreased in the FD group (baseline: 9 (15 − 4) µg/L, 9 months: 6 (13 − 4) µg/L; Suppl. Tables 3–5), however, not significant. In the KD group, Leptin decreased significantly by 3.5 (6.4) µg/L at 9 months (*p* = 0.044). Adiponectin significantly increased in the FD (+ 1.2 (2.3) µg/mL, *p* = 0.009) and with a trend in the KD group (+ 2.4 (5.4) µg/mL, *p* = 0.075). Insulin concentrations were lower at 9 months (Unstandardized B = − 1.7, *p* = 0.040) and 18 months (Unstandardized B = − 3.3, *p* = 0.001) in the FD group and at 18 months in the KD group (Unstandardized B = − 2.3, *p* = 0.058), each vs. the SD group (Suppl. Tables 3–5). Median weight losses at 9 months vs. baseline were 2.0 kg, 2.8 kg and 3.8 kg in the SD, FD, and KD group, respectively. Linear regression analysis showed that the KD (Unstandardized B = − 2.195, *p* = 0.039) and FD group (Unstandardized B = − 2.313, *p* = 0.022) had a lower body weight and BMI (KD, Unstandardized B = -0.9, *p* = 0.014) (FD, Unstandardized B = − 0.8, *p* = 0.015) compared to the SD group at 9 months, but not at 18 months. In all three groups, however, the BMI decreased significantly at 9 months (Fig. 4A). Waist circumference was 3 cm smaller at 18 months in the KD vs. the SD group (*p* = 0.044) and decreased within the KD group over time (Fig. 4B). In line with this, body fat mass (Unstandardized B = − 1.653, *p* = 0.015) and abdominal fat mass (Unstandardized B = − 2.2, *p* = 0.008) were lower in the KD vs. the SD group at 9 months, but not at 18 months. In the KD group, abdominal fat mass decreased from 31% at baseline to 26% at 9 and 28% at 18 months. Body cell mass (Unstandardized B = − 1.3, *p* = 0.022) and fat free mass (Unstandardized B = − 1.2, *p* = 0.047) were lower in the FD vs. the SD group at 9 months, but not 18 months. Systolic blood pressure decreased in the SD group (Fig. 4E), while diastolic blood pressure decreased at different time points in the KD and FD group (Fig. 4F).

#### Dietary intake

Food records revealed no relevant macro- or micronutrient intake deficiencies in the FD and KD group. The KD group reduced their carbohydrate intake from 40% at baseline to 13% and 20% of energy intake at 9 and 18 months, respectively. Their fat intake increased from 41% at baseline to 65% and 58% of energy intake at 9 and 18 months, respectively. Fiber intake increased in the SD group at 9 months vs. baseline by (+ 9 [18] g/d up to 33 [19] g/d, *p* = 0.028) and was higher than in the FD (20 [11] g/d) and the KD group (25 [8] g/d). At 18 months, fiber intake in the FD group (22 [10] g/d) was still lower than in the SD (30 [10] g/d) and KD group (27 [10] g/d). Comprehensive overviews on dietary intake (macro and micronutrient intake) in all participants and diet groups are displayed in Suppl. Tables 6–9.

#### Safety of the diets and compliance

Eighty-one participants completed the study in the Full Analysis Set (FAS) population, in which all exploratory analyses of secondary endpoints were performed.

All diets were well tolerated, and adverse events were mostly mild and transient (total, *n* = 311; SD, *n* = 77; FD, *n* = 115; KD, *n* = 119). The portion of the groups and types of adverse events can be found in Suppl. Table 1. Serious adverse events were not related to the diets (total, *n* = 11; SD, *n* = 2; FD, *n* = 2; KD, *n* = 7; Suppl. Table 2).

**Fig. 5:**
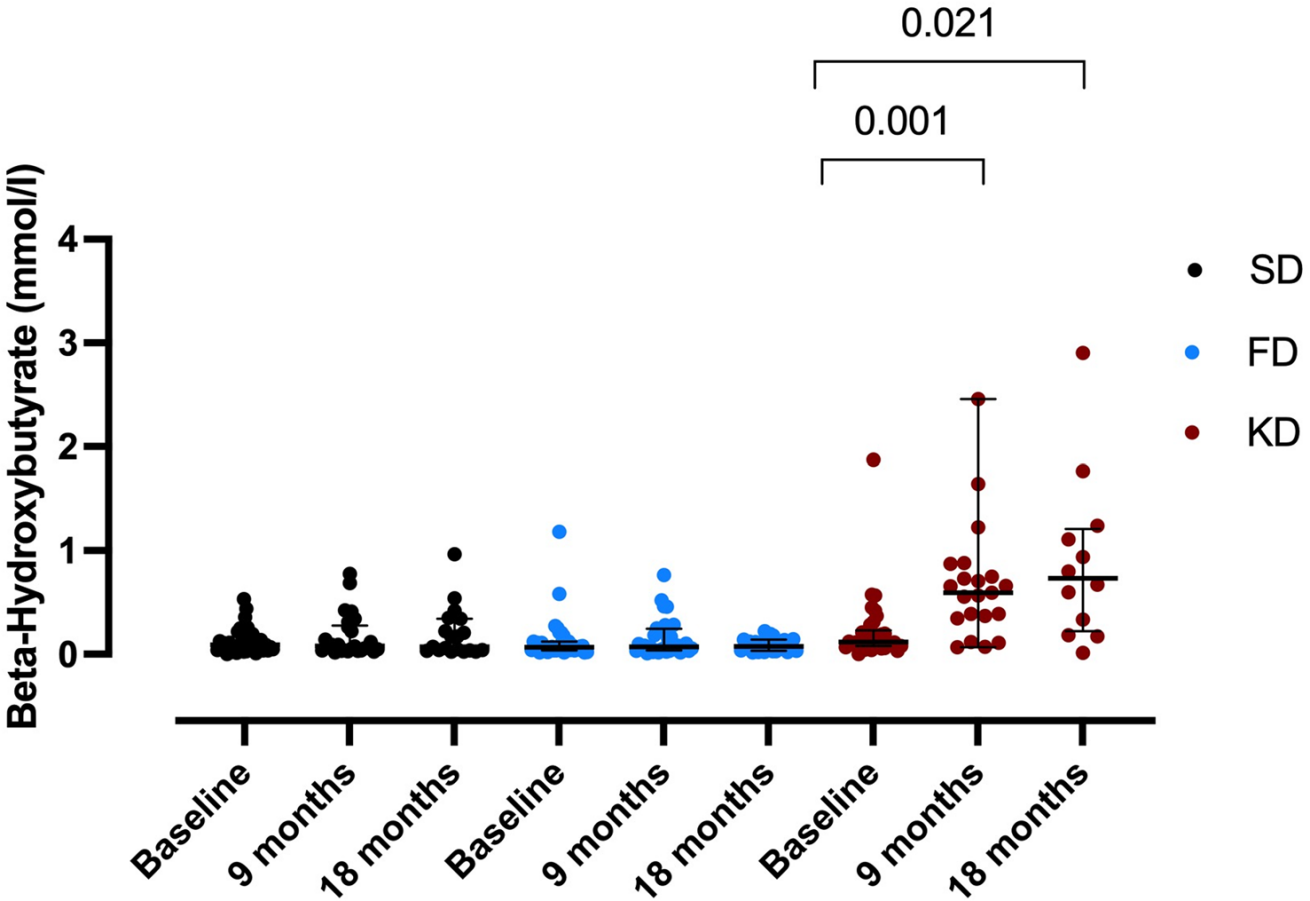
Adherence in the standard diet (SD, black circles), fasting diet (FD, blue circles) and ketogenic diet (KD, red circles) of the NAMS study. **A**) Blood beta-hydroxybutyrate concentrations confirmed nutritional ketosis in the KD group (FAS population). Data as median and interquartile range, *p* values by Wilcoxon signed-rank test

Attendance rates of dietary sessions were 88%, 100% and 77% in the SD, FD, and KD group, respectively. In the KD group, plasma BHB was ≥ 0.5 mmol/L in 62% of participants at 9 months and in 35% at 18 months, with median concentrations ≥ 0.5 mmol/L, indicating nutritional ketosis on group level at both time points (Fig. 5).

#### Predictors and determinants for disease outcomes

Predictors for having at least one new lesion at 18 months could not be identified. BHB blood concentrations were not associated with improved cognition (linear regression analysis, *p* = 0.734). However, there was a trend for an inverse association between triglyceride concentrations at 9 months and the SDMT score at 18 months in the KD group only (linear regression analysis, adjusted R-square: 0.178, Unstandardized B: − 0.328, *p* = 0.064). Additionally, there was an inverse correlation between diastolic blood pressure and SDMT scores at 18 months (Pearson correlation, *r* = − 0.396, *p* = 0.050) in the KD group, which could be reproduced as a trend in a linear regression (Unstandardized B = − 0.646, *p* = 0.054). Controlling for body weight, the trend remains (linear regression, Unstandardized B = − 0.626, *p* = 0.069).

The BDI-II score at 18 months was positively associated with body weight at 9 months in the FD group (trend, linear regression, Unstandardized B = 0.231, *p* = 0.054). Additionally, there was a positive correlation for leptin levels and BDI-II-scores at 18 months in the FD group (linear regression, Unstandardized B = 0.507, *p* = 0.043), a correlation that remained borderline significant when controlling for body weight (linear regression, Unstandardized B = 0.431, *p* = 0.059).

## Discussion

To our knowledge the NAMS study is the largest randomized-controlled study investigating in MS ketone-based diets (ketogenic and fasting diet) in comparison to a standard healthy diet.

### Primary outcome, MRI outcomes and neurodegeneration markers

We report no disease progression in terms of new cranial MRI lesions in any of the groups after 18 months study duration. Although a recent cohort study reported a relationship of pro-inflammatory diets with lesion volume and relapse rates [33], an anti-inflammatory dietary intervention did not show effects on MRI outcomes in a RCT [4]. In contrast, a small randomized-controlled pilot study did show effects of 12-weeks intermittent caloric restriction on cortical volume and thickness [34]. Even though we could not show an additional advantage of FD and KD, the complete lack of new lesions is a favorable result, especially considering the recruitment of people with MS with verified active disease (defined as one new lesion or relapse within the last two years prior to enrolment). In a comparable MS population, within 2 years, there was an increase of 17 new lesions in the placebo group compared to 3–4 new lesions under dimethyl fumarate [35]. Noteworthy, 45% of our participants were on stable DMTs at study inclusion. This leads to a reduction in disease activity per se, which subsequently makes it more difficult to detect any influence on disease activity. However, including participants on different DMTs and no DMTs was unavoidable to ensure recruitment in a reasonable time frame and it certainly reflects real world conditions [18].

We found a decrease of NfL in the FD group at 9 months. As NfL concentrations correlated well with several MS disease outcomes (EDSS, lesions count, cognition) in our cohort and others [36, 37], this decrease may suggest decelerated axonal injury due to fasting [38]. However, subjects with high BHB levels were reported to have a higher degree of improvement in serum NfL concentrations on KD in a study [39].

### Patient-centered outcomes

Depressive symptoms, cognitive impairment and fatigue are highly prevalent in MS and may have detrimental effects on patient’s autonomy, socioeconomic status and quality of life [40]. KDs have been shown to improve fatigue in MS, however, the lack of a control group [17] or very small sample sizes [41] limit inferences. Our study participants anecdotally described being less fatigued and more attentive while on KD. Despite, this was not reflected in the FSS scores of the overall groups. This discrepancy might be because the FSS rather queries physical fatigue than mental fatigue. Lower mental fatigue may be reflected by improved SDMT scores (+ 3.7 points at 18 months) in our KD group, a change considered clinically relevant [42]. There were slight effects on depressive symptoms in the FD group.

A clinically relevant change of the BDI-II score is reported by a reduction of more than 18% [43]. Our FD group improved by 22%, while our SD group deteriorated by 29%. This is in line with previous data showing improved domains of depressive symptoms due to fasting [44]. In our FD group, body weight was positively associated with depression scores, indicating that this effect might have been mediated by weight reduction. Similar results were reported by Fitzgerald et al. [9]. Of note, the BDI-II does not seem to be best suitable for assessing depressive symptoms during dietary interventions, because it queries changes of appetite and sleep, which may be desirable side effects, e.g. in a KD [45, 46]. Besides, when interpreting our results, it must be considered that depressive symptoms were only minimally prevalent at baseline in our cohort.

### Cardiometabolic health, anthropometry and body composition

Elevated leptin concentrations are prevalent in MS [47], even independently of BMI [48], and are associated with reduced regulatory T-cells [49] as well as with more severe disability, also independently of BMI [50]. In our KD group, leptin decreased, and adiponectin increased in the KD and FD group. In addition, we found a positive association between leptin and depression scores in the FD group, indicating a positive effect of leptin reduction, which has been postulated previously [51]. Our diets had different, but overall beneficial effects on all measured blood lipids, which have been shown repeatedly to be associated with cognition in MS cohorts [52–55]. In addition, NfL concentrations and HDL cholesterol were shown to be inversely correlated, suggesting metabolic alterations to be contributors to MS disease course [56]. Our diets improved markers of cardiometabolic risk, such as body weight and composition, lipid profile, adipokines and blood pressure that are not only prevalent in MS, but also associated with an adverse disease course of MS [57–59]. Especially hypertension affects grey matter atrophy and white matter damage [60]. In line with Motl et al. [61], diastolic blood pressure, which was reduced by 5 mmHg at 18 months in our KD group, was inversely correlated with cognition scores. Vascular comorbidities have been shown to be associated with cognitive dysfunction in MS [62], suggesting a beneficial effect of blood pressure reduction due to dietary interventions.

### Dietary intake, safety of the diets and compliance

We show that these dietary approaches are safe with no relevant nutrient deficiencies, and only mild, transient adverse events. Repeated periodic fasting combined with time-restricted eating seemed to be more feasible than continuous reduction of carbohydrate intake, as evidenced by smaller dropout rates in the FD vs. KD group and less adherence in the KD at 18 months. Participants reported that the KD required drastic changes in daily life, much discipline, was time-consuming and socially less acceptable. Anecdotal observations suggest that the challenges posed by the COVID-19 pandemic – such as lockdowns and travel restrictions - added to the psychological burden for patients following an already restrictive diet like the KD. Better adherence to rather time-restricted eating compared to continuous caloric restriction has been shown previously [63]. Adverse events were more frequent in the FD and KD group, however, that may have been biased by increased awareness due to detailed explanation of possible side effects of KD and FD or more frequent meetings in the FD group.

## Limitations

We planned this study with 111 participants (expected dropout rate 10%) and included 105 participants with an actual overall dropout rate of 21%. We could not show a superiority of KD or FD diets compared to a healthy control diet: we suggest that the healthy standard diet of the control group has already a relevant influence on the disease activity, that it was not inferior to the ketone-based diets. However, for ethical reasons it was reasonable to offer the control group at least the standard dietary treatment, especially over a period of 18 months. Furthermore, even though the overall study population is one of the largest nutritional intervention studies in MS, the group size of each intervention was rather small. Therefore, considering the heterogeneous disease course of our patients and the use of different DMTs of MS, our results need to be replicated in larger and more homogenous follow-up studies. In our study only a modest peripheral ketosis was measured and the full potential of ketone bodies as signaling molecules in the brain has probably not been achieved. In contrast to our study, measurements of ketones in a rather continuous manner such as via using ketone sensors measuring ketone every 15 min in the interstitial, may be more insightful to understand important fluctuations of ketosis and individual responses to foods.

Of note, a part of the study took place during the COVID-19 pandemic and was therefore severely affected by travel and contact restrictions, necessitating the change from in-person to online nutritional counselling, which may have affected motivation of study participants leading to higher dropout rates and slowed down recruitment. Additional effects of this turbulent time on our study outcomes such as on mental health cannot be ruled out. As dietary interventions are by nature unblinded, we aimed to minimize placebo effects by not communicating any interim results to participants, However, placebo effects, especially regarding patient-reported outcomes cannot be ruled out.

## Conclusion

The three group-based dietary interventions examined in this study were associated with a stable MS disease course and improvements in cardiometabolic health. A trend was observed suggesting that the ketogenic diet may positively influence cognitive function, while the fasting diet may alleviate depressive symptoms. Overall, the findings suggest beneficial effects of dietary interventions, supporting their potential role as complementary strategies in the management of RRMS. These results warrant further investigation in larger and more homogeneous study populations to allow for definitive conclusions.

## Supplementary Information

Below is the link to the electronic supplementary material.

**Supplementary Material 1**: **Suppl. Table 1**. Adverse events in the Intention-to-Treat population of the NAMS study. **Suppl. Table 2.** Serious adverse events in the Intention-to-Treat population of the NAMS study. **Suppl. Table 3.** Outcome data of the NAMS study at baseline in the Full Analysis Set (FAS). **Suppl. Table 4.** Outcome data of the NAMS at 9 months in the Full Analysis Set (FAS). **Suppl. Table 5.** Outcome data of the NAMS at 18 months in the Full Analysis Set (FAS). **Suppl. Table 6.** Dietary intake data for all participants. **Suppl. Table 7.** Dietary intake data for standard diet group. **Suppl. Table 8.** Dietary intake data for fasting diet group. **Suppl. Table 9.** Dietary intake data for ketogenic diet group. **Suppl. Table 10.** CONSERVE Checklist.

Supplementary Material 2

## Data Availability

The datasets used and analysed during the current study available from the corresponding author on reasonable request. Requests can be directed to lina.bahr@charite.de.

## Abbreviations

BDI: Beck depression inventory
BHB: Beta-hydroxybutyrate
BMI: Body mass index
DMT: Disease modifying therapy
EAE: Experimental autoimmune encephalomyelitis
EDSS: Expanded disability status scale
FAS: Full analysis set
FSS: Fatigue severity scale
FD: Fasting diet
ITT: Intention-to-treat
IQR: Interquartile range
KD: Ketogenic diet
MRI: Magnetic resonance imaging
MS: Multiple sclerosis
MSFC: Multiple sclerosis functional composite
NAMS: Nutritional approaches in multiple Sclerosis
NfL: Neurofilament light chain
PP: Per protocol
QoL: Quality of life
RRMS: Relapsing-remitting multiple sclerosis
SD: Standard diet
SDMT: Symbol digit modalities test
StD: Standard deviation

## References

[1] Wekerle H. Nature, nurture, and microbes: the development of multiple sclerosis. Acta Neurol Scand. 2017;136(Suppl 201):22–5. 29068487 10.1111/ane.12843

[2] Mische LJ, Mowry EM. The evidence for dietary interventions and nutritional supplements as treatment options in multiple sclerosis: a review. Curr Treat Options Neurol. 2018;20(4):8. 29549521 10.1007/s11940-018-0494-5

[3] Mousavi-Shirazi-Fard Z, Mazloom Z, Izadi S, Fararouei M. The effects of modified anti-inflammatory diet on fatigue, quality of life, and inflammatory biomarkers in relapsing-remitting multiple sclerosis patients: a randomized clinical trial. Int J Neurosci. 2021;131(7):657–65. 32249637 10.1080/00207454.2020.1750398

[4] Yadav V, Marracci G, Kim E, Spain R, Cameron M, Overs S, Riddehough A, Li DK, McDougall J, Lovera J, Murchison C, Bourdette D. Low-fat, plant-based diet in multiple sclerosis: A randomized controlled trial. Mult Scler Relat Disord. 2016;9:80–90. 27645350 10.1016/j.msard.2016.07.001

[5] Storoni M, Plant GT. The therapeutic potential of the ketogenic diet in treating progressive multiple sclerosis. Mult Scler Int. 2015;2015:681289. 26839705 10.1155/2015/681289 PMC4709725

[6] Piccio L, Stark JL, Cross AH. Chronic calorie restriction attenuates experimental autoimmune encephalomyelitis. J Leukoc Biol. 2008;84(4):940–8. 18678605 10.1189/jlb.0208133 PMC2638732

[7] Kafami L, Raza M, Razavi A, Mirshafiey A, Movahedian M, Khorramizadeh MR. Intermittent feeding attenuates clinical course of experimental autoimmune encephalomyelitis in C57BL/6 mice. Avicenna J Med Biotechnol. 2010;2(1):47–52. 23407146 PMC3558143

[8] Razeghi Jahromi S, Ghaemi A, Alizadeh A, Sabetghadam F, Moradi Tabriz H, Togha M. Effects of intermittent fasting on experimental autoimune encephalomyelitis in C57BL/6 mice. Iran J Allergy Asthma Immunol. 2016;15(3):212–9. 27424136

[9] Fitzgerald KC, Vizthum D, Henry-Barron B, Schweitzer A, Cassard SD, Kossoff E, Hartman AL, Kapogiannis D, Sullivan P, Baer DJ, Mattson MP, Appel LJ, Mowry EM. Effect of intermittent vs. daily calorie restriction on changes in weight and patient-reported outcomes in people with multiple sclerosis. Mult Scler Relat Disord. 2018;23:33–9. 29753994 10.1016/j.msard.2018.05.002 PMC6107078

[10] Winesett SP, Bessone SK, Kossoff EH. The ketogenic diet in pharmacoresistant childhood epilepsy. Expert Rev Neurother. 2015;15(6):621–8. 25994046 10.1586/14737175.2015.1044982

[11] Kossoff EH. More fat and fewer seizures: dietary therapies for epilepsy. Lancet Neurol. 2004;3(7):415–20. 15207798 10.1016/S1474-4422(04)00807-5

[12] Reger MA, Henderson ST, Hale C, Cholerton B, Baker LD, Watson GS, Hyde K, Chapman D, Craft S. Effects of beta-hydroxybutyrate on cognition in memory-impaired adults. Neurobiol Aging. 2004;25(3):311–4. 15123336 10.1016/S0197-4580(03)00087-3

[13] Vanitallie TB, Nonas C, Di Rocco A, Boyar K, Hyams K, Heymsfield SB. Treatment of Parkinson disease with diet-induced hyperketonemia: a feasibility study. Neurology. 2005;64(4):728–30. 15728303 10.1212/01.WNL.0000152046.11390.45

[14] Kim DY, Hao J, Liu R, Turner G, Shi FD, Rho JM. Inflammation-mediated memory dysfunction and effects of a ketogenic diet in a murine model of multiple sclerosis. PLoS ONE. 2012;7(5):e35476. 22567104 10.1371/journal.pone.0035476 PMC3342287

[15] Choi IY, Piccio L, Childress P, Bollman B, Ghosh A, Brandhorst S, Suarez J, Michalsen A, Cross AH, Morgan TE, Wei M, Paul F, Bock M, Longo VD. A diet mimicking fasting promotes regeneration and reduces autoimmunity and multiple sclerosis symptoms. Cell Rep. 2016;15(10):2136–46. 27239035 10.1016/j.celrep.2016.05.009 PMC4899145

[16] Brenton JN, Banwell B, Bergqvist AGC, Lehner-Gulotta D, Gampper L, Leytham E, Coleman R, Goldman MD. Pilot study of a ketogenic diet in relapsing-remitting MS. Neurol Neuroimmunol Neuroinflamm. 2019;6(4):e565. 31089482 10.1212/NXI.0000000000000565 PMC6487505

[17] Brenton JN, Lehner-Gulotta D, Woolbright E, Banwell B, Bergqvist AGC, Chen S, Coleman R, Conaway M, Goldman MD. Phase II study of ketogenic diets in relapsing multiple sclerosis: safety, tolerability and potential clinical benefits. J Neurol Neurosurg Psychiatry. 2022;93(6):637–44. 35418509 10.1136/jnnp-2022-329074 PMC9350909

[18] Bahr LS, Bock M, Liebscher D, Bellmann-Strobl J, Franz L, Pruss A, Schumann D, Piper SK, Kessler CS, Steckhan N, Michalsen A, Paul F, Mahler A. Ketogenic diet and fasting diet as nutritional approaches in multiple sclerosis (NAMS): protocol of a randomized controlled study. Trials. 2020;21(1):3. 31898518 10.1186/s13063-019-3928-9 PMC6941322

[19] Bahr LS. Effects of fasting and a ketogenic diet on neuropsychiatric outcomes in multiple sclerosis patients – a randomized controlled trial. Charié - Universitätsmedizin Berlin 2022.

[20] Thompson AJ, Banwell BL, Barkhof F, Carroll WM, Coetzee T, Comi G, Correale J, Fazekas F, Filippi M, Freedman MS, Fujihara K, Galetta SL, Hartung HP, Kappos L, Lublin FD, Marrie RA, Miller AE, Miller DH, Montalban X, Mowry EM, Sorensen PS, Tintore M, Traboulsee AL, Trojano M, Uitdehaag BMJ, Vukusic S, Waubant E, Weinshenker BG, Reingold SC, Cohen JA. Diagnosis of multiple sclerosis: 2017 revisions of the McDonald criteria. Lancet Neurol. 2018;17(2):162–73. 29275977 10.1016/S1474-4422(17)30470-2

[21] Wilhelmi de Toledo F, Buchinger A, Burggrabe H, Holz G, Kuhn C, Lischka E, Lischka N, Lutzner H, May W, Ritzmann-Widderich M, Stange R, Wessel A, Boschmann M, Peper E, Michalsen A. Medical association for F, nutrition. Fasting therapy - an expert panel update of the 2002 consensus guidelines. Forsch Komplementmed. 2013;20(6):434–43. 24434758 10.1159/000357602

[22] Hamilton GF, McDonald C, Chenier TC. Measurement of grip strength: validity and reliability of the sphygmomanometer and Jamar grip dynamometer. J Orthop Sports Phys Ther. 1992;16(5):215–9. 18796752 10.2519/jospt.1992.16.5.215

[23] Goldman MD, Marrie RA, Cohen JA. Evaluation of the six-minute walk in multiple sclerosis subjects and healthy controls. Mult Scler. 2008;14(3):383–90. 17942508 10.1177/1352458507082607

[24] Fischer JS, Rudick RA, Cutter GR, Reingold SC. The multiple sclerosis functional composite measure (MSFC): an integrated approach to MS clinical outcome assessment. National MS society clinical outcomes assessment task force. Mult Scler. 1999;5(4):244–50. 10467383 10.1177/135245859900500409

[25] Smith A. Symbol digit modalities test (Revised). Western Psychological Services. 1982.

[26] Aaron T, Beck RAS, Gregory K. Brown. Beck Depression Inventory – 2nd Edition (BDI-II)1996.

[27] Wintjen L, Petermann F. Beck-Depressions-Inventar revision (BDI-II). Z Psychiatr Psych Ps. 2010;58(3):243–5.

[28] Krupp LB, LaRocca NG, Muir-Nash J, Steinberg AD. The fatigue severity scale. Application to patients with multiple sclerosis and systemic lupus erythematosus. Arch Neurol. 1989;46(10):1121–3. 2803071 10.1001/archneur.1989.00520460115022

[29] Vickrey BG, Hays RD, Harooni R, Myers LW, Ellison GW. A health-related quality of life measure for multiple sclerosis. Qual Life Res. 1995;4(3):187–206. 7613530 10.1007/BF02260859

[30] Harris PA, Taylor R, Minor BL, Elliott V, Fernandez M, O’Neal L, McLeod L, Delacqua G, Delacqua F, Kirby J, Duda SN, Consortium RE. The REDCap consortium: Building an international community of software platform partners. J Biomed Inf. 2019;95:103208. 10.1016/j.jbi.2019.103208 PMC7254481 31078660

[31] Harris PA, Taylor R, Thielke R, Payne J, Gonzalez N, Conde JG. Research electronic data capture (REDCap)--a metadata-driven methodology and workflow process for providing translational research informatics support. J Biomed Inf. 2009;42(2):377–81. 10.1016/j.jbi.2008.08.010 PMC2700030 18929686

[32] R: A language and environment for statistical computing. R Foundation for Statistical Computing, Vienna, Austria. 2023 [Available from: https://www.R-project.org/

[33] Saul AM, Taylor BV, Blizzard L, Simpson-Yap S, Oddy WH, Shivappa N, Hébert JR, Black LJ, Ponsonby AL, Broadley SA, Lechner-Scott J, van der Mei I, Investigators AA. A pro-inflammatory diet in people with multiple sclerosis is associated with an increased rate of relapse and increased FLAIR lesion volume on MRI in early multiple sclerosis: A prospective cohort study. Mult Scler J. 2023;29(8):1012–23. 10.1177/13524585231167739 PMC10338707 37148166

[34] Rahmani F, Ghezzi L, Tosti V, Liu J, Song SK, Wu AT, Rajamanickam J, Obert KA, Benzinger TLS, Mittendorfer B, Piccio L, Raji CA. Twelve weeks of intermittent caloric restriction diet mitigates neuroinflammation in midlife individuals with multiple sclerosis: A pilot study with implications for prevention of alzheimer’s disease. J Alzheimers Dis. 2023;93(1):263–73. 37005885 10.3233/JAD-221007 PMC10460547

[35] Gold R, Kappos L, Arnold DL, Bar-Or A, Giovannoni G, Selmaj K, Tornatore C, Sweetser MT, Yang M, Sheikh SI, Dawson KT, Investigators DS. Placebo-controlled phase 3 study of oral BG-12 for relapsing multiple sclerosis. N Engl J Med. 2012;367(12):1098–107. 22992073 10.1056/NEJMoa1114287

[36] Kuhle J, Plavina T, Barro C, Disanto G, Sangurdekar D, Singh CM, de Moor C, Engle B, Kieseier BC, Fisher E, Kappos L, Rudick RA, Goyal J. Neurofilament light levels are associated with long-term outcomes in multiple sclerosis. Mult Scler. 2020;26(13):1691–9. 31680621 10.1177/1352458519885613 PMC7604552

[37] Harris S, Comi G, Cree BAC, Arnold DL, Steinman L, Sheffield JK, Southworth H, Kappos L, Cohen JA, Investigators OS. Plasma neurofilament light chain concentrations as a biomarker of clinical and radiologic outcomes in relapsing multiple sclerosis: post hoc analysis of phase 3 ozanimod trials. Eur J Neurol. 2021;28(11):3722–30. 34292643 10.1111/ene.15009 PMC9291872

[38] Kuhle J, Kropshofer H, Haering DA, Kundu U, Meinert R, Barro C, Dahlke F, Tomic D, Leppert D, Kappos L. Blood neurofilament light chain as a biomarker of MS disease activity and treatment response. Neurology. 2019;92(10):E1007–15. 30737333 10.1212/WNL.0000000000007032 PMC6442011

[39] Oh U, Woolbright E, Lehner-Gulotta D, Coleman R, Conaway M, Goldman MD, Brenton JN. Serum neurofilament light chain in relapsing multiple sclerosis patients on a ketogenic diet. Mult Scler Relat Disord. 2023;73:104670. 36996634 10.1016/j.msard.2023.104670 PMC10239314

[40] Silveira C, Guedes R, Maia D, Curral R, Coelho R. Neuropsychiatric symptoms of multiple sclerosis: state of the Art. Psychiatry Investig. 2019;16(12):877–88. 31805761 10.30773/pi.2019.0106 PMC6933139

[41] Lee JE, Titcomb TJ, Bisht B, Rubenstein LM, Louison R, Wahls TL. A modified MCT-Based ketogenic diet increases plasma beta-Hydroxybutyrate but has less effect on fatigue and quality of life in people with multiple sclerosis compared to a modified paleolithic diet: A Waitlist-Controlled, randomized pilot study. J Am Coll Nutr. 2020;40:1–13. 10.1080/07315724.2020.1734988 PMC11833548 32213121

[42] Benedict RH, Cohan S, Lynch SG, Riester K, Wang P, Castro-Borrero W, Elkins J, Sabatella G. Improved cognitive outcomes in patients with relapsing-remitting multiple sclerosis treated with daclizumab beta: results from the DECIDE study. Mult Scler. 2018;24(6):795–804. 28485186 10.1177/1352458517707345 PMC5971365

[43] Button KS, Kounali D, Thomas L, Wiles NJ, Peters TJ, Welton NJ, Ades AE, Lewis G. Minimal clinically important difference on the Beck depression Inventory–II according to the patient’s perspective. Psychol Med. 2015;45(15):3269–79. 26165748 10.1017/S0033291715001270 PMC4611356

[44] Fond G, Macgregor A, Leboyer M, Michalsen A. Fasting in mood disorders: neurobiology and effectiveness. A review of the literature. Psychiatry Res. 2013;209(3):253–8. 23332541 10.1016/j.psychres.2012.12.018

[45] Gibson AA, Seimon RV, Lee CM, Ayre J, Franklin J, Markovic TP, Caterson ID, Sainsbury A. Do ketogenic diets really suppress appetite? A systematic review and meta-analysis. Obes Rev. 2015;16(1):64–76. 25402637 10.1111/obr.12230

[46] Michalsen A, Schlegel F, Rodenbeck A, Ludtke R, Huether G, Teschler H, Dobos GJ. Effects of short-term modified fasting on sleep patterns and daytime vigilance in non-obese subjects: results of a pilot study. Ann Nutr Metab. 2003;47(5):194–200. 12748412 10.1159/000070485

[47] Górska E, Tylicka M, Hermanowicz A, Matuszczak E, Sankiewicz A, Gorodkiewicz E, Hermanowicz J, Karpinska E, Socha K, Kochanowicz J, Jakoniuk M, Kaminska J, Homsak E, Koper-Lenkiewicz OM. UCHL1, besides leptin and fibronectin, also could be a sensitive marker of the relapsing-remitting type of multiple sclerosis. Sci Rep-Uk. 2023;13(1). 10.1038/s41598-023-30237-3 PMC9974955 36854961

[48] Moharami S, Nourazarian A, Nikanfar M, Laghousi D, Shademan B, Joodi Khanghah O, Khaki-Khatibi F. Investigation of serum levels of orexin-A, transforming growth factor beta, and leptin in patients with multiple sclerosis. J Clin Lab Anal. 2022;36(1):e24170. 34894407 10.1002/jcla.24170 PMC8761413

[49] Matarese G, Carrieri PB, La Cava A, Perna F, Sanna V, De Rosa V, Aufiero D, Fontana S, Zappacosta S. Leptin increase in multiple sclerosis associates with reduced number of CD4(+)CD25 + regulatory T cells. Proc Natl Acad Sci U S A. 2005;102(14):5150–5. 15788534 10.1073/pnas.0408995102 PMC555982

[50] Loonstra FC, Falize KF, de Ruiter LRJ, Schoonheim MM, Strijbis EMM, Killestein J, de Vries HE, Uitdehaag BMJ, Rijnsburger M. Adipokines in multiple sclerosis patients are related to clinical and radiological measures. J Neurol. 2023;270(4):2018–30. 36562851 10.1007/s00415-022-11519-8 PMC10025234

[51] Igwe O, Sone M, Matveychuk D, Baker GB, Dursun SM. A review of effects of calorie restriction and fasting with potential relevance to depression. Prog Neuropsychopharmacol Biol Psychiatry. 2021;111:110206. 33316333 10.1016/j.pnpbp.2020.110206

[52] Siddiqui K, Browne RW, Benedict RHB, Jakimovski D, Weinstock-Guttman B, Zivadinov R, Ramanathan M. Cholesterol pathway biomarkers are associated with neuropsychological measures in multiple sclerosis. Mult Scler Relat Disord. 2023;69:104374. 36403378 10.1016/j.msard.2022.104374

[53] Andaloro A, Russo M, Pastura C, Sessa E, Calatozzo P, Maggio MG, Bramanti P. Is there a correlation between dyslipidemia and cognitive impairment in patients with multiple sclerosis? Int J Neurosci. 2020;132. 10.1080/00207454.2020.1807980 32767908

[54] Noori H, Gheini MR, Rezaeimanesh N, Saeedi R, Aliabadi HR, Sahraian MA, Moghadasi AN. The correlation between dyslipidemia and cognitive impairment in multiple sclerosis patients. Mult Scler Relat Dis. 2019;36. 10.1016/j.msard.2019.101415 31586799

[55] Hernandez-Ledesma AL, Rodriguez-Mendez AJ, Gallardo-Vidal LS, Garcia-Gasca T, Alatorre-Cruz JM, Garcia-Solis P, Lopez Reyes J, Solis-Sainz JC. Lipid profile: causal relationship on cognitive performance in multiple sclerosis? Mol Biol Rep. 2020;47(12):9667–76. 33259011 10.1007/s11033-020-06011-3

[56] Silva AS, Guimaraes J, Sousa C, Mendonca L, Soares-Dos-Reis R, Mendonca T, Abreu P, Sequeira L, Sa MJ. Metabolic syndrome parameters and multiple sclerosis disease outcomes: A Portuguese cross-sectional study. Mult Scler Relat Disord. 2023;69:104370. 36401965 10.1016/j.msard.2022.104370

[57] Oliveira SR, Simao AN, Kallaur AP, de Almeida ER, Morimoto HK, Lopes J, Dichi I, Kaimen-Maciel DR, Reiche EM. Disability in patients with multiple sclerosis: influence of insulin resistance, adiposity, and oxidative stress. Nutrition. 2014;30(3):268–73. 24484677 10.1016/j.nut.2013.08.001

[58] Penesova A, Vlcek M, Imrich R, Vernerova L, Marko A, Meskova M, Grunnerova L, Turcani P, Jezova D, Kollar B. Hyperinsulinemia in newly diagnosed patients with multiple sclerosis. Metab Brain Dis. 2015;30(4):895–901. 25809135 10.1007/s11011-015-9665-1

[59] Zhornitsky S, McKay KA, Metz LM, Teunissen CE, Rangachari M. Cholesterol and markers of cholesterol turnover in multiple sclerosis: relationship with disease outcomes. Mult Scler Relat Disord. 2016;5:53–65. 26856944 10.1016/j.msard.2015.10.005

[60] Dossi DE, Chaves H, Heck ES, Rodriguez Murua S, Ventrice F, Bakshi R, Quintana FJ, Correale J, Farez MF. Effects of systolic blood pressure on brain integrity in multiple sclerosis. Front Neurol. 2018;9:487. 29988562 10.3389/fneur.2018.00487 PMC6026666

[61] Motl RW, Baird JF, Sandroff BM, Baynard T, Fernhall B. Blood pressure and cognition in older adults with multiple sclerosis: preliminary examination. Neurol Sci. 2023;44(2):677–83. 36287283 10.1007/s10072-022-06466-1

[62] Marrie RA, Patel R, Figley CR, Kornelsen J, Bolton JM, Graff LA, Mazerolle EL, Helmick C, Uddin MN, Figley TD, Marriott JJ, Bernstein CN, Fisk JD. Effects of vascular comorbidity on cognition in multiple sclerosis are partially mediated by changes in brain structure. Front Neurol. 2022;13:910014. 35685743 10.3389/fneur.2022.910014 PMC9170886

[63] Roman SN, Fitzgerald KC, Beier M, Mowry EM. Safety and feasibility of various fasting -mimicking diets among people with multiple sclerosis. Mult Scler Relat Dis. 2020;42. 10.1016/j.msard.2020.102149 32408153

