# Supplementary material for: Fasting, ketogenic, and anti-inflammatory diets in multiple sclerosis: a randomized controlled trial with 18-month follow-up
Source: BMC Nutr. 2025 Aug 20;11:167. doi: 10.1186/s40795-025-01156-5 (PMC12366206; doi:10.1186/s40795-025-01156-5)
Supplement: Supplementary file 1 — Supplementary Material 1: Suppl. Table 1. Adverse events in the Intention-to-Treat population of the NAMS study. Suppl. Table 2. Serious adverse events in the Intention-to-Treat population of the NAMS study. Suppl. Table 3. Outcome data of the NAMS study at baseline in the Full Analysis Set (FAS). Suppl. Table 4. Outcome data of the NAMS at 9 months in the Full Analysis Set (FAS). Suppl. Table 5. Outcome data of the NAMS at 18 months in the Full Analysis Set (FAS). Suppl. Table 6. Dietary intake data for all participants. Suppl. Table 7. Dietary intake data for standard diet group. Suppl. Table 8. Dietary intake data for fasting diet group. Suppl. Table 9. Dietary intake data for ketogenic diet group. Suppl. Table 10. CONSERVE Checklist. [file 40795_2025_1156_MOESM1_ESM.docx]

**Fasting, ketogenic and anti-inflammatory diets stabilized active relapsing-remitting multiple sclerosis over 18 months – a randomized, controlled study**

**Supplement**

**Suppl. table 1*.*** Adverse events in the Intention-to-Treat population of the NAMS study

**Suppl. table 2.** Serious adverse events in the Intention-to-Treat population of the NAMS study

**Suppl. table 3.** Outcome data of the NAMS study at baseline in the Full Analysis Set (FAS)

**Suppl. table 4.** Outcome data of the NAMS at 9 months in the Full Analysis Set (FAS)

**Suppl. table 5.** Outcome data of the NAMS at 18 months in the Full Analysis Set (FAS)

**Suppl. table 6.** Dietary intake data for all participants

**Suppl. table 7.** Dietary intake data for standard diet group

**Suppl. table 8.** Dietary intake data for fasting diet group

**Suppl. table 9.** Dietary intake data for ketogenic diet group

**Supplemental table 1*.*** Adverse events in the Intention-to-Treat population of the NAMS study

| **Adverse events (%)** | **All** | **SD** | **FD** | **KD** |
| --- | --- | --- | --- | --- |
| Sample size (n) | 105 | 34 | 35 | 36 |
| General diseases and complaints at the administration site | 27 | 15 | 26 | 39 |
| Eye diseases | 4 | 0 | 9 | 3 |
| Endocrine diseases | 6 | 0 | 9 | 8 |
| Diseases of the respiratory, thoracic and mediastinum | 16 | 21 | 11 | 17 |
| Diseases of the skin and subcutaneous tissue | 10 | 3 | 11 | 14 |
| Diseases of the kidney and urinary tract | 9 | 9 | 9 | 8 |
| Diseases of the blood and lymphatic system | 16 | 15 | 17 | 17 |
| Gastrointestinal diseases | 25 | 21 | 34 | 19 |
| Diseases of the immune system (including allergies) | 7 | 9 | 3 | 8 |
| Diseases of the nervous system | 21 | 12 | 31 | 19 |
| Diseases of the ear and labyrinth | 6 | 3 | 9 | 6 |
| Vascular diseases | 2 | 0 | 3 | 3 |
| Benign, malignant, unspecified neoplasm (including cysts and polyps) | 3 | 3 | 3 | 3 |
| Heart diseases | 4 | 0 | 6 | 6 |
| Infection and parasitic diseases | 57 | 47 | 69 | 56 |
| Liver and gallbladder diseases | 3 | 0 | 0 | 8 |
| Psychiatric disorders | 7 | 12 | 0 | 8 |
| Musculoskeletal, connective tissue and bone disorders | 34 | 29 | 43 | 31 |
| Metabolic and nutritional disorders | 16 | 9 | 17 | 22 |
| Examinations | 2 | 3 | 3 | 0 |
| Injury, poisoning, and procedure-related complications | 7 | 3 | 6 | 11 |
| Pregnancy, childbirth, and perinatal diseases | 7 | 6 | 3 | 11 |
| Surgical and medical procedures | 10 | 9 | 6 | 14 |
| Social circumstances | 1 | 0 | 3 | 0 |

FD, fasting diet; KD, ketogenic diet; SD, standard diet

**Supplemental table 2.** Serious adverse events in the Intention-to-Treat population of the NAMS study

| **Serious adverse events (n)** | **SD** | **FD** | **KD** |
| --- | --- | --- | --- |
| Imminently life-threatening | 1 | 0 | 1 |
| Hospital stay or extension | 1 | 1 | 5 |
| Coronavirus disease (COVID-19) | 0 | 1 | 1 |

FD, fasting diet; KD, ketogenic diet; SD, standard diet

**Supplemental table 3.** Outcome data of the NAMS study at baseline in the Full Analysis Set (FAS)

| FAS Baseline | All (n) | mean (StD) | median (Q3-Q1) | KD (n) | mean (StD) | median (Q3-Q1) | FD (n) | mean (StD) | median (Q3-Q1) | SD (n) | mean (StD) | median (Q3-Q1) |
| --- | --- | --- | --- | --- | --- | --- | --- | --- | --- | --- | --- | --- |
| Age (years) | 81 | 43 (10) | 44 (50-33) | 26 | 42 (10) | 43 (51-32) | 30 | 42  (9) | 43 (50-35) | 25 | 44 (10) | 46 (51-35) |
| EDSS score | 71 | 2.1 (1.0) | 2.0 (2.5-1.5) | 24 | 2.2 (1.2) | 2.0 (3.1-1.5) | 24 | 1.9 (0.9) | 2.0 (2.5-1.5) | 23 | 2.1 (0.8) | 2.0 (2.5-1.5) |
| T2 lesions, number (n) | 81 | 55.3 (58.1) | 34.0 (76.0-18.0) | 26 | 48.2 (58.3) | 29.0 (50.3-15.8) | 30 | 48.8 (38.1) | 39.0 (59.8-20.3) | 25 | 70.8 (74.7) | 36.0 (88.0-19.0) |
| T2 lesions, volume (mm^3^) | 81 | 6.7 (8.9) | 3.5 (8.4-1.2) | 26 | 7.0 (10.8) | 2.2 (7.7-1.0) | 30 | 6.4 (6.6) | 4.4 (9.6-1.5) | 25 | 6.9 (9.5) | 3.5 (7.3-1.5) |
| FSS score | 80 | 3.7 (1.8) | 3.7 (5.3-2.1) | 26 | 3.5 (1.6) | 3.5 (4.8-2.2) | 29 | 3.9 (1.8) | 3.9 (5.2-2.6) | 25 | 3.7 (1.9) | 3.8 (5.3-1.8) |
| BDI-II score | 81 | 9.0 (8.0) | 8.0 (12.0-3.0) | 26 | 8.7 (8.7) | 6.0 (10.8-3.0) | 30 | 9.5 (7.4) | 9.0 (13.0-5.3) | 25 | 8.5 (8.1) | 7.0 (12.0-2.0) |
| SDMT score | 77 | 56.0 (11.3) | 56.0 (62.0-49.0) | 25 | 55.1 (13.3) | 57.0 (65.0-47.0) | 27 | 56.0 (10.0) | 56.0 (61.5-49.0) | 25 | 56.8 (11.0) | 54.0 (62.0-51.0) |
| 6-MWT (min) | 79 | 558.3 (102.1) | 566.5 (616.2-502.5) | 25 | 556.0(94.4) | 564.1 (608.1-487.6) | 29 | 559.2 (124.7) | 561.5 (638.1-502.7) | 25 | 559.6 (82.5) | 570 (609.8-510.8) |
| Hand grip strength (kg) | 78 | 33.6 (9.1) | 32.5 (37.1-27.6) | 26 | 35.2 (10.3) | 33.6 (38.6-27.6) | 28 | 32.0 (8.9) | 31.8 (36.4-25.9) | 24 | 33.9 (8.0) | 32.1 (36.6-27.8) |
| MSQOL54 Physical Health score | 80 | 69.6 (14.5) | 71.3 (78.5-63.4) | 25 | 68.3 (12.8) | 70.1 (77.6-65.9) | 30 | 70.7 (12.6) | 71.8 (76.6-65.8) | 25 | 69.5 (18.2) | 70.3 (85.2-54.2) |
| MSQOL54 Mental Health score | 80 | 68.1 (15.7) | 74.2 (79.9-57.7) | 25 | 65.7 (16.2) | 73.6 (79.8-57.5) | 30 | 68.5 (14.8) | 74.2 (78.8-59.2) | 25 | 67.0 (16.7) | 76.8 (83.1-57.8) |
| Average MSFC hpt dominant hand (sec) | 79 | 20.5 (3.4) | 19.8 (21.9-18.5) | 25 | 20.3 (3.9) | 19.3 (20.8-18.3) | 29 | 20.4 (2.6) | 19.9 (21.6-18.6) | 25 | 21.0 (3.7) | 20.8 (22.0-19.2) |
| Average MSFC t25fw (sec) | 78 | 4.3 (0.7) | 4.2 (4.7-3.8) | 25 | 4.3 (0.7) | 4.1 (4.8-3.9) | 28 | 4.3 (0.8) | 4.4 (4.7-3.9) | 25 | 4.1 (0.6) | 4.1 (4.5-3.7) |
| Z Score t25fw | 78 | 0.0 (1.1) | 0.1 (0.6-(-0.5)) | 25 | -0.1 (1.1) | 0.2 (0.4-(-0.9)) | 28 | -0.1 (1.2) | -0.1 (0.6-(-0.7)) | 25 | 0.2 (0.9) | 0.2 (0.8-(-0.3)) |
| Z Score PASAT | 78 | 0.1 (0.8) | 0.3 (0.8-(-0.4)) | 24 | 0.2 (0.7) | 0.2 (0.825-(-0.3)) | 29 | -0.2 (0.8) | -0.3 (0.6-(-0.6)) | 25 | 0.4 (0.7) | 0.6 (0.9-0.2) |
| Z Score hpt | 79 | -0.1 (1.0) | 0.0 (0.5-(-0.6)) | 25 | 0.1 (1.1) | 0.2 (0.56-(-0.2)) | 29 | -0.1 (0.8) | 0.0 (0.5-(-0.4)) | 25 | -0.2 (1.1) | -0.3 (0.3-(-0.7)) |
| Overall Z Score | 77 | 0.0 (0.7) | 0.2 (0.5-(-0.3)) | 24 | 0.1 (0.7) | 0.3 (0.6-(-0.2)) | 28 | -0.1 (0.7) | -0.1 (0.3-(-0.4)) | 25 | 0.1 (0.7) | 0.2 (0.6-(-0.1)) |
| Disease duration from first manifestation (years) | 81 | 9.2 (7.7) | 6.7 (14.8-2.3) | 26 | 10.4 (8.1) | 8.6 (16.5-2.9) | 30 | 9.0 (6.7) | 8.5 (14.6-4.2) | 25 | 8.2 (8.4) | 3.2 (12.7-1.9) |
| Disease duration from first diagnosis (years) | 80 | 6.1 (5.9) | 3.6 (10.1-1.4) | 26 | 6.4 (6.5) | 4.6 (9.6-1.3) | 30 | 7.0 (5.4) | 5.5 (12.0-2.1) | 24 | 4.5 (5.8) | 1.8 (5.1-1.1) |
| BMI (kg/m^2^) | 81 | 24.9 (4.1) | 24.3 (26.3-22.3) | 26 | 25.0 (5.2) | 23.7 (25.8-22.2) | 30 | 24.3 (3.7) | 23.7 (25.9-21.5) | 25 | 25.5 (3.4) | 25.0 (27.1-23.7) |
| Body fat (%) | 78 | 29.6 (8.1) | 29.7 (34.4-24.7) | 25 | 29.5 (9.9) | 29.1 (32.5-24.1) | 29 | 29.0 (6.8) | 29.3 (33.5-24.9) | 24 | 30.2 (7.7) | 32.3 (34.6-25.9) |
| Total cholesterol (mg/dL) | 67 | 192 (35) | 188 (212-173) | 22 | 187.6 (36.3) | 182 (205-156) | 22 | 195 (36) | 196 (214-176) | 23 | 193 (33) | 188 (206-179) |
| LDL cholesterol (mg/dL) | 67 | 119 (30) | 113 (142-97) | 22 | 119 (32) | 119 (145-93) | 22 | 117 (32) | 109 (132-97) | 23 | 122 (28) | 113 (137-106) |
| HDL cholesterol (mg/dL) | 67 | 70 (17) | 71 (80-57) | 22 | 69 (16) | 71 (79-54) | 22 | 74 (19) | 79 (83-57) | 23 | 69 (14) | 68 (79-62) |
| Triglycerides (mg/dL) | 62 | 88 (40) | 77 (100-66) | 22 | 85 (37) | 77 (84-66) | 19 | 96 (39) | 91 (118-67) | 21 | 85 (44) | 70 (98-66) |
| Insulin (mU/L) | 81 | 8.5 (5.1) | 7.0 (9.5-5.8) | 26 | 8.9 (6.1) | 6.5 (8.6-6.1) | 30 | 8.0 (4.5) | 7.0 (9.3-5.1) | 25 | 8.8 (4.7) | 7.4 (9.6-5.9) |
| Leptin (μg/L) | 75 | 11.3 (8.8) | 9.6 (15.0-5.1) | 22 | 11.8 (10.9) | 9.5 (14.3-5.2) | 28 | 10.3 (8.7) | 8.6 (14.2-4.2) | 25 | 11.9 (6.9) | 9.7 (17.3-6.9) |
| Adiponectin (μg/mL) | 79 | 9.5 (5.1) | 8.4 (12.1-5.6) | 24 | 10.1 (4.8) | 8.9 (13.2-6.3) | 30 | 9.1 (5.5) | 8.7 (11.4-4.5) | 25 | 9.4 (5.1) | 8.0 (11.7-6.3) |
| Vitamin D (nmol/L) | 79 | 101.6 (35.9) | 97.9 (119.6-79.5) | 25 | 103.2 (33.4) | 97.9 (117.3-86.6) | 29 | 93.8 (35.7) | 92.2 (119.5-70.1) | 25 | 108.9 (38.2) | 103.9 (125.7-82.1) |
| Beta-hydroxybutyrate (mmol/L) | 79 | 0.14 (0.18) | 0.09 (0.17-0.04) | 24 | 0.19 (0.17) | 0.11 (0.23-0.08) | 30 | 0.13 (0.23) | 0.07 (0.12-0.04) | 25 | 0.11 (0.12) | 0.07 (0.15-0.04) |
| NfL (pg/mL) | 55 | 11.5 (7.1) | 9.8 (12.8-7.3) | 12 | 12.3 (6.7) | 10.2 (14.3-8.1) | 25 | 12.5 (8.7) | 10.1 (13.7-7.2) | 17 | 10.0 (4.5) | 9.1 (11.6-7.3) |

FD, fasting diet; KD, ketogenic diet; SD, standard diet

**Supplemental table 4.** Outcome data of the NAMS at 9 months in the Full Analysis Set (FAS)

| FAS 9 months | All (n) | mean (StD) | median (Q3-Q1) | KD (n) | mean (StD) | median (Q3-Q1) | FD (n) | mean (StD) | median (Q3-Q1) | SD (n) | mean (StD) | median (Q3-Q1) |
| --- | --- | --- | --- | --- | --- | --- | --- | --- | --- | --- | --- | --- |
| T2 lesions, number (n) | 79 | 57 (58) | 35 (78-20) | 24 | 51 (61) | 30 (56-17) | 30 | 50 (39) | 41 (59-21) | 25 | 70.1 (72) | 36 (87-20) |
| T2 lesions, volume (mm^3^) | 79 | 7.3  (9.7) | 3.9 (9.5-1.3) | 24 | 8.1  (12.1) | 2.7 (9.5-1.1) | 30 | 6.7  (6.8) | 4.72 (9.9-1.56) | 25 | 7.4  (10.4) | 3.6 (8.2-1.5) |
| Brain atrophy measured by percentage of brain volume change | 79 | -0.3 (0.5) | -0.3 (0.1-(-0.6)) | 24 | -0.4 (0.5) | -0.4 (-0.2-(-0.7)) | 30 | -0.2 (0.5) | -0.2 (0.1-(-0.4)) | 25 | -0.2 (0.4) | -0.2 (0.2-(-0.5)) |
| FSS score | 79 | 3.8 (1.7) | 3.8 (5.1-2.4) | 25 | 3.6 (1.6) | 3.3 (4.6-2.4) | 29 | 4.0 (1.7) | 4.4 (5.0-2.7) | 25 | 3.9 (1.8) | 3.7 (5.6-2.2) |
| BDI-II score | 81 | 9.8 (9.4) | 8.0 (14.0-3.0) | 26 | 10.3 (12.4) | 5.5 (14.8-2.0) | 30 | 8.2 (7.2) | 7.5 (12.8-3.0) | 25 | 11.2 (8.1) | 10.0 (15.0-8.0) |
| SDMT score | 79 | 56.7 (12.2) | 57.0 (66.0-47.5) | 24 | 58.0 (14.9) | 60.0 (71.0-47.8) | 30 | 56.4 (11.6) | 56.5 (63.8-50.3) | 25 | 55.9 (10.5) | 57.0 (65.0-47.0) |
| 6-MWT (min) | 78 | 567.9 (85.4) | 580.0 (629.4-518.5) | 23 | 576.4 (70.9) | 582.0 (619.7-541.0) | 30 | 562.7 (100.6) | 569.2 (644.8-488.2) | 25 | 566.3 (80.5) | 572.4 (630.4-522.2) |
| Hand grip strength (kg) | 79 | 33.6 (9.5) | 32.8 (36.8-27.8) | 24 | 35.0 (9.3) | 34.1 (37.1-28.9) | 30 | 31.2 (10.3) | 31.3 (34.2-25.1) | 25 | 35.0 (8.6) | 33.8 (40.6-28.5) |
| MSQOL54 Physical Health score | 76 | 69.8 (14.8) | 72.5 (82.3-60.9) | 24 | 69.4 (14.9) | 74.1 (82.4-55.8) | 28 | 70.2 (13.9) | 68.8 (78.4-62.1) | 24 | 69.8 (16.3) | 73.6 (84.2-57.9) |
| MSQOL54 Mental Health score | 77 | 66.0 (16.8) | 69.7 (80.1-57.2) | 25 | 64.5 (19.6) | 68.2 (81.2-53.0) | 27 | 66.6 (16.6) | 70.0 (77.8-58.8) | 25 | 67.0 (14.4) | 69.7 (77.8-59.5) |
| Average MSFC hpt dominant hand (sec) | 79 | 20.3 (3.2) | 19.7 (21.5-17.9) | 24 | 19.8 (3.1) | 19.5 (21.1-17.0) | 30 | 20.72 (3.4) | 19.76 (23.1-18.1) | 25 | 20.3 (3.1) | 19.7 (20.8-18.5) |
| Average MSFC t25fw (sec) | 78 | 4.5 (2.1) | 4.2 (4.7-3.8) | 23 | 4.2 (0.6) | 4.2 (4.6-3.8) | 30 | 4.3 (0.7) | 4.2 (4.8-3.8) | 25 | 4.9 (3.5) | 4.2 (4.6-3.9) |
| Z Score t25fw | 78 | -0.4 (3.2) | 0.2 (0.7-(-0.8)) | 23 | 0.1 (1.0) | 0.2 (0.7-(-0.6)) | 30 | -0.1 (1.1) | 0.0 (0-7-(-0.9)) | 25 | -1.0 (5.4) | 0.2 (0.5-(-0.6)) |
| Z Score PASAT | 78 | 0.1 (0.9) | 0.3 (0.7-(-0.4)) | 23 | 0.2 (0.7) | 0.4 (0.7-(-0.2)) | 30 | -0.3 (1.0) | -0.3 (0.5-(-0.9)) | 25 | 0.3 (0.8) | 0.6 (0.9-(-0.1)) |
| Z Score hpt | 79 | 0.0 (1.0) | 0.1 (0.7-(-0.5)) | 24 | 0.2 (1.0) | 0.2 (1.1-(-0.4)) | 30 | -0.1 (1.0) | 0.0 (0.7-(-0.8)) | 25 | -0.0 (0.9) | 0.0 (0.5-(-0.3)) |
| Overall Z Score | 77 | -0.1 (1.2) | 0.1 (0.5-(-0.3)) | 22 | 0.2 (0.6) | 0.3 (0.7-(-0.2)) | 30 | -0.2 (0.7) | -0.1 (0.3-(-0.6)) | 25 | -0.2 (1.9) | 0.2 (0.5-(-0.2)) |
| Disease duration from first manifestation (years) | 81 | 9.2 (7.7) | 6.7 (14.8-2.3) | 26 | 10.4 (8.1) | 8.6 (16.5-2.9) | 30 | 9.0 (6.7) | 8.5 (4.2-14.6) | 25 | 8.2 (8.4) | 3.2 (12.7-1.9) |
| Disease duration from first diagnosis (years) | 80 | 6.1 (5.9) | 3.6 (10.1-1.4) | 26 | 6.4 (6.5) | 4.6 (9.6-1.3) | 30 | 7.0 (5.4) | 5.5 (12.0-2.1) | 24 | 4.5 (5.8) | 1.8 (5.1-1.1) |
| BMI (kg/m^2^) | 78 | 24.0 (2.9) | 23.6 (25.5-22.3) | 23 | 23.4 (2.5) | 22.9 (24.6-22.5) | 30 | 23.5 (2.8) | 23.1 (25.1-21.6) | 25 | 25.1 (3.3) | 24.9 (26.6-23.1) |
| Body fat (%) | 79 | 27.7 (7.0) | 27.9 (32.3-24.3) | 24 | 26.4 (8.3) | 27.1 (30.8-23.4) | 30 | 27.6 (5.9) | 27.2 (31.7-24.3) | 25 | 29.0 (6.8) | 30.9 (33.7-25.9) |
| Total cholesterol (mg/dL) | 70 | 185 (37) | 182 (208-160) | 20 | 189 (40) | 178 (203-164) | 29 | 186 (40) | 187 (211-147) | 21 | 179 (30) | 175 (194-156) |
| LDL cholesterol (mg/dL) | 71 | 111 (33) | 109 (129-90) | 20 | 118 (34) | 112 (132-98) | 29 | 108 (36) | 100 (126-85) | 22 | 109 (28) | 109 (123-91) |
| HDL cholesterol (mg/dL) | 71 | 71 (20) | 72 (81-59) | 20 | 75 (24) | 77 (83-64) | 29 | 75 (20) | 79 (83-59) | 22 | 63 (13) | 66 (71-54) |
| Triglycerides (mg/dL) | 69 | 78 (32) | 70 (89-54) | 20 | 61 (18) | 53 (72-48) | 29 | 85 (33) | 75 (109-62) | 20 | 85 (36) | 79 (92-69) |
| Insulin (mU/L) | 69 | 7.7 (3.7) | 6.9 (9.8-5.1) | 19 | 7.6 (2.9) | 7.1 (9.0-5.8) | 30 | 7.0 (3.3) | 6.7 (8.7-4.4) | 20 | 9.0 (4.5) | 7.5 (12.4-5.3) |
| Leptin (μg/L) | 68 | 8.8 (5.2) | 7.8 (12.4-4.5) | 17 | 7.2 (3.2) | 7.5 (8.5-4.5) | 29 | 8.7 (5.9) | 5.9 (12.6-4.5) | 22 | 10.3 (5.3) | 10.8 (14.3-5.7) |
| Adiponectin (μg/mL) | 71 | 10.8 (5.9) | 9.7 (13.0-6.7) | 19 | 13.1 (6.9) | 12.4 (17.1-8.2) | 30 | 10.3 (5.9) | 9.9 (11.8-6.0) | 22 | 9.3 (4.2) | 9.0 (10.9-6.8) |
| Vitamin D (nmol/L) | 68 | 110.0 (38.8) | 108.5 (130.6-83.4) | 19 | 124.8 (44.5) | 113.6 (133.7-96.1) | 29 | 96.3 (36.4) | 94.8 (116.9-75.8) | 20 | 115.9 (30.5) | 114.3 (139.9-93.3) |
| Beta-hydroxybutyrate (mmol/L) | 72 | 0.31 (0.41) | 0.11 (0.46-0.04) | 20 | 0.69 (0.57) | 0.62 (0.78-0.37) | 30 | 0.16 (0.18) | 0.07 (0.25-0.04) | 22 | 0.18 (0.22) | 0.08 (0.29-0.04) |
| NfL (pg/mL) | 55 | 10.3 (4.4) | 8.9 (12.1-7.5) | 12 | 10.4 (4.5) | 10.1 (13.0-6.2) | 25 | 10.4 (5.0) | 8.2 (14.5-7.2) | 18 | 10.0 (3.6) | 8.9 (10.8-7.8) |

**Supplemental table 5.** Outcome data of the NAMS at 18 months in the Full Analysis Set (FAS)

| FAS 18 months | All (n) | mean (StD) | median (Q3-Q1) | KD (n) | mean (StD) | median (Q3-Q1) | FD (n) | mean (StD) | median (Q3-Q1) | SD (n) | mean (StD) | median (Q3-Q1) |
| --- | --- | --- | --- | --- | --- | --- | --- | --- | --- | --- | --- | --- |
| T2 lesions, number (n) | 79 | 55 (57) | 35 (68-20) | 25 | 48 (59) | 28 (45-16) | 30 | 50 (39) | 41 (63-22) | 24 | 70 (71) | 37 (101-20) |
| T2 lesions, volume (mm^3^) | 79 | 7.5 (10.4) | 3.6 (9.5-1.3) | 25 | 8.0 (12.4) | 2.7 (9.4-1.2) | 30 | 7.0 (7.1) | 5.0 (11.1-1.6) | 24 | 7.8 (11.8) | 3.3 (8.8-1.4) |
| Brain atrophy measured by percentage of brain volume change | 79 | -0.6 (0.8) | -0.5 (-0.1-(-0.8)) | 25 | -0.7 (0.8) | -0.7 (-0.2-(-1.0)) | 30 | -0.4 (0.8) | -0.2 (0.2-(-0.7)) | 24 | -0.6 (0.6) | -0.5 (-0.2-(-0.7)) |
| FSS score | 81 | 3.9 (1.8) | 4.1 (5.3-2.2) | 26 | 3.8 (1.7) | 3.6 (5.1-2.7) | 30 | 4.1 (1.8) | 4.8 (5.6-2.7) | 25 | 3.6 (1.9) | 3.6 (4.9-1.8) |
| BDI-II score | 81 | 9.3 (9.7) | 7.0 (13.0-2.0) | 26 | 9.7 (9.8) | 6.0 (11.8-3.3) | 30 | 7.5 (7.7) | 7.0 (10.5-2.0) | 25 | 11.0 (11.4) | 9.0 (14.0-3.0) |
| SDMT score | 81 | 58.0 (11.8) | 57.0 (68.0-48.0) | 26 | 59.4 (13.3) | 57.5 (73.0-48.0) | 30 | 57.7 (10.7) | 57.5 (67.3-49.0) | 25 | 56.8 (11.6) | 57.0 (64.0-49.0) |
| 6-MWT (min) | 81 | 559.2 (90.4) | 578.0 (625.3-485.0) | 26 | 552.0 (96.5) | 576.3 (600.6-509.4) | 30 | 558.7 (87.2) | 561.3 (620.9-502.3) | 25 | 567.4 (91.2) | 593.5 (636.0-517.3) |
| Hand grip strength (kg) | 78 | 33.8 (9.4) | 32.6 (37.1-27.8) | 24 | 35.7 (38.5-28.2) | 36.1 (38.5-28.2) | 29 | 31.5 (10.1) | 31.2 (34.6-25.9) | 25 | 34.7 (7.8) | 33.6 (37.6-28.5) |
| MSQOL54 Physical Health score | 79 | 70.4 (16.8) | 76.3 (81.9-59.7) | 25 | 69.4 (15.5) | 75.3 (80.5-56.6) | 29 | 72.2 (14.6) | 76.0 (81.9-66.5) | 25 | 69.3 (20.6) | 79.3 (84.2-50.2) |
| MSQOL54 Mental Health score | 81 | 67.3 (16.6) | 71.3 (80.5-58.9) | 26 | 65.9 (17.1) | 70.2 (80.7-52.5) | 30 | 67.8 (15.2) | 70.0 (80.1-58.9) | 25 | 68.1 (18.2) | 73.7 (80.5-62.2) |
| Average MSFC hpt dominant hand (sec) | 80 | 20.1 (3.3) | 19.5 (21.1-17.7) | 25 | 19.4 (3.1) | 18.9 (20.2-17.7) | 30 | 20.9 (3.5) | 20.7 (21.8-18.5) | 25 | 19.8 (3.2) | 19.3 (21.0-17.7) |
| Average MSFC t25fw (sec) | 81 | 4.4 (0.7) | 4.2 (4.7-3.9) | 26 | 4.4 (0.9) | 4.2 (4.8-3.9) | 30 | 4.4 (0.6) | 4.3 (4.8-3.9) | 25 | 4.2 (0.5) | 4.1 (4.5-3.9) |
| Z Score t25fw | 81 | -0.2 (1.1) | 0.1 (0.6-(-0.7)) | 26 | -0.2 (1.4) | 0.2 (0.6-(-0.8)) | 30 | -0.2 (1.0) | -0.1 (0.4-(-0.8)) | 25 | -0.0 (0.8) | 0.1 (0.6-(-0.4)) |
| Z Score PASAT | 80 | 0.2 (1.0) | 0.4 (0.9-(-0.1)) | 25 | 0.2 (0.9) | 0.4 (0.8-0.1) | 30 | -0.1 (1.1) | 0.2 (0.7-(-0.6)) | 25 | 0.4 (0.8) | 0.7 (1.0-0.2) |
| New Z Score hpt | 80 | 0.1 (1.0) | 0.1 (0.8-(-0.4)) | 25 | 0.3 (1.0) | 0.3 (0.9-(-0.1)) | 30 | -0.2 (1.0) | -0.3 (0.6-(-0.6)) | 25 | 0.2 (1.0) | 0.2 (0.8-(-0.4)) |
| Overall Z Score | 79 | 0.1 (0.7) | 0.3 (0.5-(-0.3)) | 24 | 0.2 (0.8) | 0.4 (0.6-0.1) | 30 | -0.2 (0.7) | 0.0 (0.3-(-0.5)) | 25 | 0.2 (0.6) | 0.3 (0.6-(-0.2)) |
| Disease duration from first manifestation (years) | 81 | 9.2 (7.7) | 6.7 (14.8-2.3) | 26 | 10.4 (8.1) | 8.6 (16.5-2.9) | 30 | 9.0 (6.7) | 8.5 (14.6-4.2) | 25 | 8.2 (8.4) | 3.2 (12.7-1.9) |
| Disease duration from first diagnosis (years) | 80 | 6.1 (5.9) | 3.6 (10.1-1.4) | 26 | 6.4 (6.5) | 4.6 (9.6-1.3) | 30 | 7.0 (5.4) | 5.5 (12.0-2.1) | 24 | 4.5 (5.8) | 1.8 (5.1-1.1) |
| BMI (kg/m^2^) | 81 | 24.4 (3.5) | 24.3 (26.0-22.2) | 26 | 24.5 (4.4) | 24.5 (25.7-22.3) | 30 | 23.5 (2.6) | 23.4 (25.5-21.7) | 25 | 25.2 (3.3) | 24.8 (26.9-23.1) |
| Body fat (%) | 77 | 28.4 (7.4) | 30.6 (32.8-23.6) | 23 | 27.8 (9.8) | 30.8 (33.4-21.1) | 29 | 27.9 (5.7) | 28.5 (31.5-23.6) | 25 | 29.6 (6.6) | 31.2 (34.5-26.5) |
| Total cholesterol (mg/dL) | 55 | 190 (40) | 187 (211-162) | 12 | 186 (39) | 180 (211-156) | 25 | 197 (42) | 190 (228-170) | 18 | 182 (39) | 182.5 (200-152) |
| LDL cholesterol (mg/dL) | 55 | 115 (34) | 110 (135-91) | 12 | 109 (27) | 111 (122-86) | 25 | 120 (38) | 114 (140-94) | 18 | 111 (33) | 105 (130-84) |
| HDL cholesterol (mg/dL) | 55 | 71 (19) | 69 (83-57) | 12 | 74 (25) | 66 (92-54) | 25 | 75 (17) | 71 (88-64) | 18 | 62 (16) | 63 (69-53) |
| Triglycerides (mg/dL) | 54 | 80 (28) | 75 (90-63) | 12 | 71 (11) | 72 (78-65) | 25 | 82 (32) | 72 (98-58) | 17 | 85 (30) | 83 (90-67) |
| Insulin (mU/L) | 53 | 7.7 (3.8) | 6.8 (9.3-5.3) | 11 | 7.4 (2.4) | 7.1 (7.4-6.2) | 24 | 6.3 (2.7) | 5.7 (7.0-4.6) | 18 | 9.8 (4.7) | 9.3 (12.7-5.7) |
| Leptin (μg/L) | 52 | 9.7 (6.5) | 9.0 (12.4-5.0) | 10 | 9.0 (5.4) | 9.3 (12.3-4.1) | 25 | 10.0 (6.5) | 9.6 (13.7-5.0) | 17 | 9.6 (7.5) | 8.7 (11.1-4.9) |
| Adiponectin (μg/mL) | 53 | 9.8 (4.3) | 8.9 (12.2-7.0) | 11 | 11.0 (4.7) | 9.5 (14.9-7.6) | 25 | 9.7 (4.1) | 9.5 (11.2-7.0) | 17 | 9.4 (4.5) | 7.6 (12.5-6.4) |
| Vitamin D (nmol/L) | 53 | 114.7 (42.7) | 107.6 (143.5-85.0) | 11 | 126.4 (40.0) | 129.1 (152.8-88.3) | 24 | 107.5 (47.4) | 93.8 (123.5-77.7) | 18 | 117.2 (37.9) | 119.4 (139.1-92.6) |
| Beta-hydroxybutyrate (mmol/L) | 54 | 0.31 (0.51) | 0.12 (0.31-0.04) | 12 | 0.90 (0.81) | 0.74 (1.14-0.30) | 24 | 0.09 (0.06) | 0.07 (0.12-0.03) | 18 | 0.2 (0.25) | 0.07 (0.31-0.04) |
| NfL (pg/mL) | 55 | 11.3 (7.2) | 9.4 (13.8-7.0) | 12 | 13.5 (12.5) | 9.8 (16.0-6.8) | 25 | 10.6 (4.0) | 9.2 (14.1-6.9) | 18 | 10.9 (6.2) | 9.4 (11.7-7.3) |

**Supplemental table 6: Dietary intake data for all participants**

| **all data as mean (SD)** | **all (n = 77)** | |  |  | **n = 69** | |  | |  | **n = 72** | |  | |  | |
| --- | --- | --- | --- | --- | --- | --- | --- | --- | --- | --- | --- | --- | --- | --- | --- |
|  | **Baseline** | | **DACH Reference** | **Delta Reference** | **9 months** | | **DACH Reference** | | **Delta Reference** | **18 months** | | **DACH Reference** | | **Delta Reference** | |
| **Energy (kcal)** | 1821 (426) | | 2050 | -229 | 1812 (847) | | 2050 | | -238 | 1774 (502) | | 2050 | | -276 | |
| **Fat (%)** | 40 (9) | | 30 | 10 | 45 (16) | | 30 | | 15 | 45 (13) | | 30 | | 15 | |
| **Fat (g)** | 83 (33) | | 0 | 83 | 92 (48) | | 0 | | 92 | 92 (45) | | 0 | | 92 | |
| **Carbohydrate (%)** | 39 (10) | | 50 | -11 | 33 (16) | | 50 | | -17 | 34 (13) | | 50 | | -16 | |
| **Carbohydrate (g)** | 151 (78) | | 0 | 151 | 137 (177) | | 0 | | 137 | 137 (83) | | 0 | | 137 | |
| **Protein (%)** | 16 (4) | | 15 | 1 | 16 (4) | | 15 | | 1 | 16 (4) | | 15 | | 1 | |
| **Protein (g)** | 70 (21) | | 52 | 18 | 70 (25) | | 52 | | 18 | 66 (18) | | 52 | | 14 | |
| **" / bodyweight (mg/kg)** | 21.9 (130.2) | | 0.8 | 21.1 | 29.3 (167.2) | | 0.8 | | 28.5 | 13.9 (109.6) | | 0.8 | | 13.1 | |
| **Water (l)** | 2.34 (0.88) | | 2.52 | -0.18 | 2.49 (1.28) | | 2.52 | | -0.03 | 2.41 (0.92) | | 2.52 | | -0.11 | |
| **Retinolequivalent (mg)** | 1.42 (1.423) | | 0.775 | 0.645 | 2.195 (8.773) | | 0.775 | | 1.42 | 1.246 (0.648) | | 0.775 | | 0.471 | |
| **Vitamine A (Retinol) (mg)** | 0.56 (1.151) | | 0.775 | -0.215 | 0.458 (0.914) | | 0.775 | | -0.32 | 0.358 (0.205) | | 0.775 | | -0.417 | |
| **Vitamine D (Calciferol) (µg)** | 3 (4) | | 2 | 1 | 3 (3) | | 2 | | 1 | 3 (3) | | 2 | | 1 | |
| **Vitamine E (Tocopherol) (mg)** | 15 (9) | | 13 | 2 | 16 (8) | | 13 | | 3 | 16 (8) | | 13 | | 3 | |
| **Vitamine K (µg)** | 172 (126) | | 65 | 107 | 187 (152) | | 65 | | 122 | 162 (120) | | 65 | | 97 | |
| **Vitamine B1 (Thiamin) (mg)** | 1.17 (0.53) | | 1.12 | 0.05 | 1.12 (0.78) | | 1.12 | | 0.00 | 1.03 (0.32) | | 1.12 | | -0.08 | |
| **Vitamine B2 (Riboflavin) (mg)** | 1.36 (0.53) | | 1.22 | 0.14 | 1.33 (0.53) | | 1.22 | | 0.11 | 1.16 (0.34) | | 1.22 | | -0.05 | |
| **Niacinequivalent (mg)** | 28.5 (8.77) | | 13.67 | 14.83 | 28.69 (11.53) | | 13.67 | | 15.02 | 26.72 (7.58) | | 13.67 | | 13.06 | |
| **Pantothenic acid (mg)** | 4 (2) | | 5 | -1 | 4 (2) | | 5 | | 1 | 4 (1) | | 5 | | -1 | |
| **Vitamine B6 (Pyridoxin) (mg)** | 1.5 (0.6) | | 1.5 | 0 | 1.5 (1.1) | | 1.5 | | 0 | 1.3 (0.4) | | 1.5 | | -0.2 | |
| **Biotin (µg)** | 50 (24) | | 40 | 10 | 53 (48) | | 40 | | 13 | 49 (19) | | 40 | | 9 | |
| **Total Folate (mg)** | 0.3 (0.1) | | 0.3 | 0 | 0.3 (0.4) | | 0.3 | | 0 | 0.3 (0.1) | | 0.3 | | 0 | |
| **Vitamine B12 (Cobalamin) (µg)** | 4 (3) | | 4 | 0 | 4 (2) | | 4 | | 0 | 3 (2) | | 4 | | -1 | |
| **Vitamine C (Ascorbin acid) (mg)** | 123.1 (173.1) | | 102.5 | 20.6 | 141.2 (369.7) | | 102.5 | | 38.8 | 96.8 (66.4) | | 102.5 | | -5.7 | |
| **Sodium (g)** | 1.9 (0.7) | 1.5 | | 0 | | 1.7 (0.7) | 1.5 | 0.2 | | | 1.6 (0.7) | | 1.5 | | 0.1 |
| **Potassium (g)** | 2.8 (0.8) | 4 | | -1 | | 3 (4) | 4 | -1 | | | 3 (1) | | 4 | | -1 |
| **Calcium (g)** | 1 (0) | 1 | | 0 | | 1 (1) | 1 | 0 | | | 1 (0) | | 1 | | 0 |
| **Magnesium (mg)** | 0.367 (0.119) | 0.325 | | 0.042 | | 0.419 (0.265) | 0.325 | 0.09 | | | 0.401 (0.154) | | 0.325 | | 0.076 |
| **Phosphoride (mg)** | 1.2 (0.3) | 0.7 | | 0.5 | 1.3 (0.5) | | 0.7 | | 0.6 | 1.2 (0.3) | | 0.7 | | 0.5 | |
| **Iron (mg)** | 11.84 (3.7) | 11.67 | | 0.17 | | 11.92 (5) | 11.67 | 0.25 | | | 12.52 (4.45) | | 11.67 | | 0.85 |
| **Cink (mg)** | 10 (3) | 11 | | -1 | | 10 (4) | 11 | -1 | | | 9 (3) | | 11 | | -2 |
| **Copper (mg)** | 1.96 (0.71) | 1.25 | | 0.71 | | 2.09 (0.92) | 1.25 | 0.84 | | | 2.09 (0.69) | | 1.25 | | 0.84 |
| **Manganese (mg)** | 5.8 (2.9) | 3.5 | | 2.3 | | 5.76 (3.6) | 3.5 | 2.3 | | | 6.2 (3.1) | | 3.5 | | 2.7 |
| **Fluoride (mg)** | 0.93 (0.57) | 3.45 | | -2.53 | | 0.88 (0.56) | 3.45 | -2.57 | | | 0.96 (0.52) | | 3.45 | | -2.49 |
| **Iodide (mg)** | 0.104 (0.058) | 0.175 | | -0.071 | | 0.103 (0.07) | 0.175 | -0.07 | | | 0.088 (0.046) | | 0.175 | | -0.087 |
| **Fibre (g)** | 23 (10) | 30 | | -7 | | 26 (14) | 30 | -4 | | | 26 (10) | | 30 | | -4 |
| **Sucrose (%)** | 8 (4) | 10 | | -2 | | 7 (6) | 10 | -3 | | | 7 (4) | | 10 | | -3 |
| **Sucrose (beet sugar) (g)** | 38 (24) | 50 | | -12 | | 40 (112) | 50 | -10 | | | 31 (20) | | 50 | | -19 |
| **Cholesterin (g)** | 0.3 (0.2) | 0.3 | | 0 | | 0.2 (0.3) | 0.3 | -0.1 | | | 0.3 (0.9) | | 0.3 | | 0 |
| **Alcohol (g)** | 5 (8) | 0 | | 5 | | 5 (7) | 0 | 5 | | | 4 (7) | | 0 | | 4 |
| **Saturated fatty acids (%)** | 32 (14) | 33 | | -1 | | 31 (17) | 33 | -2 | | | 32 (15) | | 33 | | -1 |
| **Saturated fatty acids (g)** | 16 (5) | 0 | | 16 | | 15 (6) | 0 | 15 | | | 16 (5) | | 0 | | 16 |
| **n-3 fatty acids (%)** | 2.1 (2.7) | 0.5 | | 1.6 | | 3 (3.2) | 0.5 | 2.5 | | | 2.9 (2.8) | | 0.5 | | 2.4 |
| **n-3 fatty acids (g)** | 257 (1011) | 0 | | 257 | | 462 (2806) | 0 | 462 | | | 249 (1176) | | 0 | | 249 |
| **n-6 fatty acids (%)** | 5.9 (3.5) | 2.5 | | 3.4 | | 7.6 (3.9) | 2.5 | 5.1 | | | 7.3 (3.3) | | 2.5 | | 4.8 |
| **n-6 fatty acids (g)** | 12 (9) | 0 | | 12 | | 15(9) | 0 | 15 | | | 15 (10) | | 0 | | 15 |

DACH = Germany, Austria, Switzerland

**Supplemental table 7: Dietary intake data for standard diet group**

| **all data as mean (SD)** | **Standard diet**  **(n = 25)** |  |  | **n = 20** |  |  | **n = 21** |  |  |
| --- | --- | --- | --- | --- | --- | --- | --- | --- | --- |
|  | **Baseline** | **DACH Reference** | **Delta Reference** | **9 months** | **DACH Reference** | **Delta Reference** | **18 months** | **DACH Reference** | **Delta Reference** |
| **Energy (kcal)** | 1923 (400) | 2050 | -127 | 2042 (1301) | 2050 | -8 | 1845 (484) | 2050 | -205 |
| **Fat (%)** | 36 (6) | 30 | 6 | 35 (10) | 30 | 5 | 40 (7) | 30 | 10 |
| **Fat (g)** | 78 (22) | 0 | 78 | 72 (18) | 0 | 72 | 85 (27) | 0 | 85 |
| **Carbohydrate (%)** | 43 (7) | 50 | -7 | 44 (12) | 50 | -6 | 39 (8) | 50 | -11 |
| **Carbohydrate (g)** | 172 (89) | 0 | 172 | 211 (298) | 0 | 211 | 155 (86) | 0 | 155 |
| **Protein (%)** | 15 (3) | 15 | 0 | 15 (3) | 15 | 0 | 15(3) | 15 | 0 |
| **Protein (g)** | 73 (19) | 52 | 21 | 72 (24) | 52 | 20 | 67 (17) | 52 | 15 |
| **" / bodyweight (mg/kg)** | 1 (0.3) | 0.8 | 0.2 | 1.1 (0.5) | 0.8 | 0.3 | 1 (0.3) | 0.8 | 0.2 |
| **Water (l)** | 2.29 (0.98) | 2.52 | -0.22 | 2.94 (1.64) | 2.52 | 0.42 | 2.65 (0.78) | 2.52 | 0.14 |
| **Retinolequivalent (mg)** | 1.349 (0.664) | 0.775 | 0.574 | 5.101 (16.204) | 0.775 | 4.326 | 1.592 (0.759) | 0.775 | 0.817 |
| **Vitamine A (Retinol) (mg)** | 0.486 (0.307) | 0.775 | -0.289 | 0.253 (0.153) | 0.775 | -0.522 | 0.278 (0.17) | 0.775 | -0.497 |
| **Vitamine D (Calciferol) (µg)** | 3 (2) | 2 | 1 | 3 (2) | 2 | 1 | 3 (2) | 2 | 1 |
| **Vitamine E (Tocopherol) (mg)** | 12 (7) | 13 | -1 | 14 (9) | 13 | 1 | 19 (7) | 13 | 6 |
| **Vitamine K (µg)** | 169 (121) | 65 | 104 | 240 (205) | 65 | 175 | 201 (127) | 65 | 136 |
| **Vitamine B1 (Thiamin) (mg)** | 1.27 (0.67) | 1.12 | 0.15 | 1.49 (1.26) | 1.12 | 0.37 | 1.15 (0.28) | 1.12 | 0.03 |
| **Vitamine B2 (Riboflavin) (mg)** | 1.47 (0.6) | 1.22 | 0.26 | 1.59 (0.49) | 1.22 | 0.37 | 1.28 (0.35) | 1.22 | 0.06 |
| **Niacinequivalent (mg)** | 28.98 (9.2) | 13.67 | 15.31 | 31.32 (14.19) | 13.67 | 17.65 | 27.38 (6.74) | 13.67 | 13.71 |
| **Pantothenic acid (mg)** | 5 (2) | 5 | 0 | 6 (4) | 5 | 1 | 4 (1) | 5 | -1 |
| **Vitamine B6 (Pyridoxin) (mg)** | 1.5 (0.6) | 1.5 | 0 | 1.9 (1.8) | 1.5 | 0.4 | 1.5 (0.4) | 1.5 | 0 |
| **Biotin (µg)** | 49 (24) | 40 | 9 | 69 (82) | 40 | 29 | 48 (16) | 40 | 8 |
| **Total Folate (mg)** | 0.3 (0.1) | 0.3 | 0 | 0.5 (0.6) | 0.3 | 0.2 | 0.3 (0.1) | 0.3 | 0 |
| **Vitamine B12 (Cobalamin) (µg)** | 4 (2) | 4 | 0 | 3 (2) | 4 | -1 | 3 (1) | 4 | -1 |
| **Vitamine C (Ascorbin acid) (mg)** | 94.7 (79.1) | 102.5 | -7.8 | 264.3 (677.8) | 102.5 | 161.8 | 111 (84) | 102.5 | 8.2 |
| **Sodium (g)** | 2 (0.8) | 1.5 | 0.5 | 1.9 (0.8) | 1.5 | 0.4 | 1.7 (0.8) | 1.5 | 0.2 |
| **Potassium (g)** | 3 (1) | 4 | -1 | 5 (6) | 4 | 1 | 3 (1) | 4 | -1 |
| **Calcium (g)** | 1 (0) | 1 | 0 | 1 (1) | 1 | 0 | 1 (0) | 1 | 0 |
| **Magnesium (mg)** | 0.392 (0.117) | 0.325 | 0.067 | 0.569 (0.405) | 0.325 | 0.244 | 0.467 (0.189) | 0.325 | 0.142 |
| **Phosphoride (mg)** | 1.4 (0.4) | 0.7 | 0.7 | 1.5 (0.6) | 0.7 | 0.8 | 1.3 (0.3) | 0.7 | 0.6 |
| **Iron (mg)** | 12.35 (4.04) | 11.67 | 0.68 | 14.4 (5.92) | 11.67 | 2.79 | 14.46 (4.77) | 11.67 | 2.79 |
| **Cink (mg)** | 11 (3) | 11 | 0 | 11 (5) | 11 | 0 | 10 (2) | 11 | -1 |
| **Copper (mg)** | 1.88 (0.54) | 1.25 | 0.63 | 2.3 (1.09) | 1.25 | 1.01 | 2.25 (0.6) | 1.25 | 1 |
| **Manganese (mg)** | 5.7 (2.9) | 3.5 | 2.2 | 6.6 (3.2) | 3.5 | 3.1 | 7.2 (2.8) | 3.5 | 3.7 |
| **Fluoride (mg)** | 0.92 (0.57) | 3.45 | -2.53 | 0.95 (0.58) | 3.45 | -2.5 | 1.1 (0.58) | 3.45 | -2.35 |
| **Iodide (mg)** | 0.127 (0.074) | 0.175 | -0.048 | 0.119 (0.097) | 0.175 | -0.056 | 0.09 (0.054) | 0.175 | -0.085 |
| **Fibre (g)** | 23 (8) | 30 | -7 | 33 (19) | 30 | 3 | 30 (10) | 30 | 0 |
| **Sucrose (%)** | 9 (4) | 10 | -1 | 9 (10) | 10 | 1 | 7 (2) | 10 | -3 |
| **Sucrose (beet sugar) (g)** | 44 (31) | 50 | -6 | 76 (205) | 50 | 26 | 34 (15) | 50 | -16 |
| **Cholesterin (g)** | 0.2 (0.1) | 0.3 | -0.1 | 0.2 (0.1) | 0.3 | -0.1 | 0.5 (1.6) | 0.3 | 0.2 |
| **Alcohol (g)** | 7 (12) | 0 | 7 | 5 (6) | 0 | 5 | 6 (9) | 0 | 6 |
| **Saturated fatty acids (%)** | 33 (14) | 33 | 0 | 24 (9) | 33 | -9 | 26 (11) | 33 | -7 |
| **Saturated fatty acids (g)** | 15 (4) | 0 | 15 | 12 (5) | 0 | 12 | 12 (3) | 0 | 12 |
| **n-3 fatty acids (%)** | 2 (2.7) | 0.5 | 1.5 | 2 (2.3) | 0.5 | 1.5 | 3.4 (1.8) | 0.5 | 2.9 |
| **n-3 fatty acids (g)** | 102 (498) | 0 | 102 | 1144 (5097) | 0 | 1144 | 362 (1626) | 0 | 362 |
| **n-6 fatty acids (%)** | 4.5 (3) | 2.5 | 2 | 5.9 (2.4) | 2.5 | 3.4 | 7.4 (3.2) | 2.5 | 4.9 |
| **n-6 fatty acids (g)** | 9 (5) | 0 | 9 | 11 (4) | 0 | 11 | 16 (8) | 0 | 16 |

DACH = Germany, Austria, Switzerland

**Supplemental table 8: Dietary intake data for fasting diet group**

| **all data as mean (SD)** | **Fasting diet**  **(n = 28)** |  |  | **n = 26** |  |  | **n = 28** |  |  |
| --- | --- | --- | --- | --- | --- | --- | --- | --- | --- |
|  | **Baseline** | **DACH Reference** | **Delta Reference** | **9 months** | **DACH Reference** | **Delta Reference** | **18 months** | **DACH Reference** | **Delta Reference** |
| **Energy (kcal)** | 1738 (419) | 2050 | -313 | 1560 (537) | 2050 | -490 | 1667 (463) | 2050 | -383 |
| **Fat (%)** | 40 (11) | 30 | 10 | 38 (8) | 30 | 8 | 39 (7) | 30 | 9 |
| **Fat (g)** | 81 (35) | 0 | 81 | 67 (27) | 0 | 67 | 74 (24) | 0 | 74 |
| **Carbohydrate (%)** | 39 (11) | 50 | -11 | 41 (8) | 50 | 9 | 40 (9) | 50 | -10 |
| **Carbohydrate (g)** | 144 (71) | 0 | 144 | 150 (70) | 0 | 150 | 161 (73) | 0 | 161 |
| **Protein (%)** | 15 (3) | 15 | 0 | 15 (3) | 15 | 0 | 15 (5) | 15 | 0 |
| **Protein (g)** | 65 (20) | 52 | 13 | 56 (20) | 52 | 4 | 60 (16) | 52 | 8 |
| **" / bodyweight (mg/kg)** | 58 (213) | 0.8 | 57.6 | 75.7 (269.1) | 0.8 | 75 | 34.1 (175.8) | 0.8 | 33.3 |
| **Water (l)** | 2.48 (0.73) | 2.52 | -0.03 | 2.45 (1.03) | 2.52 | -0.07 | 2.3 (0.93) | 2.52 | -0.21 |
| **Retinolequivalent (mg)** | 1.275 (0.966) | 0.775 | 0.5 | 0.919 (0.37) | 0.775 | 0.144 | 1.076 (0.563) | 0.775 | 0.301 |
| **Vitamine A (Retinol) (mg)** | 0.413 (0.359) | 0.775 | -0.362 | 0.58 (1.46) | 0.775 | -0.195 | 0.338 (0.165) | 0.775 | -0.437 |
| **Vitamine D (Calciferol) (µg)** | 3 (4) | 2 | 1 | 2 (1) | 2 | 0 | 2 (1) | 2 | 0 |
| **Vitamine E (Tocopherol) (mg)** | 16 (9) | 13 | 3 | 13 (7) | 13 | 0 | 13 (7) | 13 | 0 |
| **Vitamine K (µg)** | 160 (100) | 65 | 95 | 138 (88) | 65 | 73 | 159 (135) | 65 | 94 |
| **Vitamine B1 (Thiamin) (mg)** | 1.11 (0.43) | 1.12 | -0.01 | 0.9 (0.37) | 1.12 | -0.22 | 1 (0.32) | 1.12 | -0.11 |
| **Vitamine B2 (Riboflavin) (mg)** | 1.17 (0.43) | 1.22 | -0.04 | 0.97 (0.36) | 1.22 | -0.25 | 1.08 (0.32) | 1.22 | -0.14 |
| **Niacinequivalent (mg)** | 27.85 (9.28) | 13.67 | 14.18 | 23.45 (8.6) | 13.67 | 9.78 | 24.38 (6.14) | 13.67 | 10.71 |
| **Pantothenic acid (mg)** | 4 (2) | 5 | -1 | 3 (1) | 5 | -2 | 4 (2) | 5 | -2 |
| **Vitamine B6 (Pyridoxin) (mg)** | 1.4 (0.4) | 1.5 | -0.1 | 1.2 (0.5) | 1.5 | -0.3 | 1.1 (0.3) | 1.5 | -0.4 |
| **Biotin (µg)** | 46 (19) | 40 | 7 | 38 (14) | 40 | -2 | 43 (17) | 40 | 3 |
| **Total Folate (mg)** | 0.3 (0.1) | 0.3 | 0 | 0.2 (0.1) | 0.3 | -0.1 | 0.3 (0.1) | 0.3 | 0 |
| **Vitamine B12 (Cobalamin) (µg)** | 4 (2) | 4 | 0 | 3 (2) | 4 | -1 | 3 (1) | 4 | -1 |
| **Vitamine C (Ascorbin acid) (mg)** | 81.3 (59.1) | 102.5 | -21.2 | 74.5 (48.8) | 102.5 | -28 | 76.8 (64.5) | 102.5 | -25.7 |
| **Sodium (g)** | 1.8 (0.8) | 1.5 | 0.3 | 1.4 (0.6) | 1.5 | -0.1 | 1.6 (0.7) | 1.5 | 0.1 |
| **Potassium (g)** | 3 (1) | 4 | -1 | 2 (1) | 4 | -2 | 2 (1) | 4 | -2 |
| **Calcium (g)** | 1 (0) | 1 | 0 | 1 (0) | 1 | 0 | 1 (0) | 1 | 0 |
| **Magnesium (mg)** | 0.341 (0.1) | 0.325 | 0.016 | 0.323 (0.142) | 0.325 | -0.002 | 0.347 (0.129) | 0.325 | 0.022 |
| **Phosphoride (mg)** | 1.1 (0.3) | 0.7 | 0.4 | 1 (0.4) | 0.7 | 0.3 | 1.1 (0.3) | 0.7 | 0.4 |
| **Iron (mg)** | 11.68 (3.25) | 11.67 | 0.01 | 10.62 (4.32) | 11.67 | -1.05 | 11.36 (3.91) | 11.67 | -0.31 |
| **Cink (mg)** | 9 (3) | 11 | -2 | 8 (3) | 11 | -3 | 8 (3) | 11 | -3 |
| **Copper (mg)** | 1.97 (0.58) | 1.25 | 0.72 | 1.84 (0.72) | 1.25 | 0.59 | 1.93 (0.7) | 1.25 | 0.68 |
| **Manganese (mg)** | 6.4 (3.4) | 3.5 | 2.9 | 5.7 (4.6) | 3.5 | 2.2 | 5.8 (3.7) | 3.5 | 2.3 |
| **Fluoride (mg)** | 1.02 (0.71) | 3.45 | -2.43 | 0.98 (0.68) | 3.45 | -2.47 | 0.95 (0.53) | 3.45 | -2.5 |
| **Iodide (mg)** | 0.088 (0.049) | 0.175 | -0.087 | 0.086 (0.048) | 0.175 | -0.089 | 0.094 (0.044) | 0.175 | -0.081 |
| **Fibre (g)** | 22 (8) | 30 | -8 | 21 (11) | 30 | -9 | 22 (7) | 30 | -8 |
| **Sucrose (%)** | 8 (4) | 10 | -2 | 8 (3) | 10 | -2 | 8 (4) | 10 | -2 |
| **Sucrose (beet sugar) (g)** | 35 (19) | 50 | -15 | 31 (19) | 50 | -19 | 36 (26) | 50 | -14 |
| **Cholesterin (g)** | 0.2 (0.3) | 0.3 | -0.1 | 0.3 (0.4) | 0.3 | 0 | 0.2 (0.1) | 0.3 | -0.1 |
| **Alcohol (g)** | 5 (6) | 0 | 5 | 7 (8) | 0 | 7 | 5 (7) | 0 | 5 |
| **Saturated fatty acids (%)** | 30 (15) | 33 | -3 | 23 (11) | 33 | -10 | 27 (10) | 33 | -6 |
| **Saturated fatty acids (g)** | 15 (5) | 0 | 15 | 13 (3) | 0 | 13 | 14 (4) | 0 | 14 |
| **n-3 fatty acids (%)** | 2.1 (2.6) | 0.5 | 1.6 | 2.7 (2.2) | 0.5 | 2.2 | 2.5 (2) | 0.5 | 2 |
| **n-3 fatty acids (g)** | 314 (1201) | 0 | 314 | 110 (534) | 0 | 110 | 5 (4) | 0 | 5 |
| **n-6 fatty acids (%)** | 6.8 (3.8) | 2.5 | 4.3 | 7.2 (4.7) | 2.5 | -2.7 | 6.5 (2.8) | 2.5 | 4 |
| **n-6 fatty acids (g)** | 14 (9) | 0 | 14 | 12 (7) | 0 | 12 | 12 (6) | 0 | 12 |

DACH = Germany, Austria, Switzerland

**Supplemental table 9: Dietary intake data for ketogenic diet group**

| **all data as mean (SD)** | **ketogenic diet**  **(n = 24)** |  |  | **n = 23** |  |  | **n = 23** |  |  |
| --- | --- | --- | --- | --- | --- | --- | --- | --- | --- |
|  | **Baseline** | **DACH Reference** | **Delta Reference** | **9 months** | **DACH Reference** | **Delta Reference** | **18 months** | **DACH Reference** | **Delta Reference** |
| **Energy (kcal)** | 1811 (455) | 2050 | -239 | 1895 (548) | 2050 | -155 | 1840 (559) | 2050 | -210 |
| **Fat (%)** | 43 (9) | 30 | 13 | 63 (10) | 30 | 33 | 56 (15) | 30 | 26 |
| **Fat (g)** | 89 (39) | 0 | 89 | 136 (52) | 0 | 136 | 121 (61) | 0 | 121 |
| **Carbohydrate (%)** | 36 (9) | 50 | -14 | 15 (10) | 50 | -35 | 23 (15) | 50 | -27 |
| **Carbohydrate (g)** | 136 (71) | 0 | 136 | 57 (50) | 0 | 57 | 93 (75) | 0 | 93 |
| **Protein (%)** | 16 (4) | 15 | 1 | 18 (4) | 15 | 3 | 17 (4) | 15 | 2 |
| **Protein (g)** | 72 (22) | 52 | 20 | 83 (25) | 52 | 31 | 73 (21) | 52 | 21 |
| **" / bodyweight (mg/kg)** | 1 (0.3) | 0.8 | 0.2 | 1.2 (0.4) | 0.8 | 0.4 | 1 (0.3) | 0.8 | 0.2 |
| **Water (l)** | 2.22 (0.96) | 2.52 | -0.3 | 2.13 (1.09) | 2.52 | -0.39 | 2.3 (1.01) | 2.52 | -0.21 |
| **Retinolequivalent (mg)** | 1.662 (2.246) | 0.775 | 0.887 | 1.113 (0.407) | 0.775 | 0.338 | 1.138 (0.529) | 0.775 | 0.363 |
| **Vitamine A (Retinol) (mg)** | 0.809 (2.007) | 0.775 | 0.034 | 0.499 (0.288) | 0.775 | -0.276 | 0.454 (0.242) | 0.775 | -0.321 |
| **Vitamine D (Calciferol) (µg)** | 4 (5) | 2 | 2 | 4 (4) | 2 | 2 | 4 (4) | 2 | 2 |
| **Vitamine E (Tocopherol) (mg)** | 15 (9) | 13 | 3 | 21 (8) | 13 | 8 | 16 (8) | 13 | 3 |
| **Vitamine K (µg)** | 190 (158) | 65 | 125 | 197 (144) | 65 | 132 | 130 (83) | 65 | 65 |
| **Vitamine B1 (Thiamin) (mg)** | 1.13 (0.49) | 1.12 | 0.02 | 1.06 (0.4) | 1.12 | -0.06 | 0.96 (0.35) | 1.12 | -0.15 |
| **Vitamine B2 (Riboflavin) (mg)** | 1.46 (0.52) | 1.22 | 0.24 | 1.51 (0.51) | 1.22 | 0.29 | 1.16 (0.33) | 1.22 | -0.06 |
| **Niacinequivalent (mg)** | 28.76 (7.99) | 13.67 | 15.09 | 32.34 (10) | 13.67 | 18.67 | 28.97 (9.22) | 13.67 | 15.31 |
| **Pantothenic acid (mg)** | 5 (2) | 5 | 0 | 4 (2) | 5 | 1 | 4 (1) | 5 | -1 |
| **Vitamine B6 (Pyridoxin) (mg)** | 1.5 (0.7) | 1.5 | 0 | 1.5 (0.4) | 1.5 | 0 | 1.3 (0.3) | 1.5 | -0.2 |
| **Biotin (µg)** | 55 (28) | 40 | 15 | 56 (20) | 40 | 16 | 57 (22) | 40 | 17 |
| **Total Folate (mg)** | 0.3 (0.2) | 0.3 | 0 | 0.3 (0.1) | 0.3 | 0 | 0.3 (0.1) | 0.3 | 0 |
| **Vitamine B12 (Cobalamin) (µg)** | 5 (4) | 4 | 1 | 5 (2) | 4 | 1 | 4 (2) | 4 | 0 |
| **Vitamine C (Ascorbin acid) (mg)** | 201.3 (281) | 102.5 | 98.8 | 109.7 (53.3) | 102.5 | 7.2 | 108.6 (43.5) | 102.5 | 6.1 |
| **Sodium (g)** | 1.9 (0.6) | 1.5 | 0.4 | 1.8 (0.7) | 1.5 | 0.3 | 1.4 (0.8) | 1.5 | -0.1 |
| **Potassium (g)** | 3 (1) | 4 | -1 | 3 (1) | 4 | -1 | 3 (1) | 4 | -1 |
| **Calcium (g)** | 1 (0) | 1 | 0 | 1 (0) | 1 | 0 | 1 (0) | 1 | 0 |
| **Magnesium (mg)** | 0.369 (0.139) | 0.325 | 0.044 | 0.396 (0.142) | 0.325 | 0.071 | 0.405 (0.128) | 0.325 | 0.08 |
| **Phosphoride (mg)** | 1.2 (0.3) | 0.7 | 0.5 | 1.4 (0.5) | 0.7 | 0.7 | 1.2 (0.3) | 0.7 | 0.5 |
| **Iron (mg)** | 11.5 (3.91) | 11.67 | -0.17 | 11.23 (4.19) | 11.67 | -0.49 | 12.16 (4.35) | 11.67 | 0.49 |
| **Cink (mg)** | 10 (3) | 11 | -1 | 11 (4) | 11 | 0 | 10 (3) | 11 | -1 |
| **Copper (mg)** | 2.03 (0.97) | 1.25 | 0.78 | 2.23 (0.93) | 1.25 | 0.98 | 2.14 (0.74) | 1.25 | 0.89 |
| **Manganese (mg)** | 5 (2.2) | 3.5 | 1.5 | 5.1 (2.3) | 3.5 | 1.6 | 5.7 (2.5) | 3.5 | 2.2 |
| **Fluoride (mg)** | 0.82 (0.34) | 3.45 | -2.63 | 0.71 (0.37) | 3.45 | -2.74 | 0.85 (0.45) | 3.45 | -2.6 |
| **Iodide (mg)** | 0.097 (0.042) | 0.175 | -0.078 | 0.109 (0.061) | 0.175 | -0.066 | 0.078 (0.04) | 0.175 | -0.097 |
| **Fibre (g)** | 25 (13) | 30 | -5 | 24 (8) | 30 | -6 | 27 (10) | 30 | -3 |
| **Sucrose (%)** | 8 (3) | 10 | -2 | 4 (2) | 10 | -6 | 5 (3) | 10 | -5 |
| **Sucrose (beet sugar) (g)** | 35 (18) | 50 | -15 | 19 (13) | 50 | -31 | 22 (13) | 50 | -28 |
| **Cholesterin (g)** | 0.3 (0.1) | 0.3 | 0 | 0.3 (0.2) | 0.3 | 0 | 0.3 (0.1) | 0.3 | 0 |
| **Alcohol (g)** | 5 (6) | 0 | 5 | 2 (4) | 0 | 2 | 1 (3) | 0 | 1 |
| **Saturated fatty acids (%)** | 34 (12) | 33 | 1 | 46 (19) | 33 | 13 | 42 (19) | 33 | 9 |
| **Saturated fatty acids (g)** | 17 (5) | 0 | 17 | 21 (5) | 0 | 21 | 20 (5) | 0 | 20 |
| **n-3 fatty acids (%)** | 2.1 (2.7) | 0.5 | 1.6 | 4.3 (4.3) | 0.5 | 3.9 | 3 (4.1) | 0.5 | 2.5 |
| **n-3 fatty acids (g)** | 351 (1177) | 0 | 351 | 268 (985) | 0 | 268 | 444 (1388) | 0 | 444 |
| **n-6 fatty acids (%)** | 6.4 (3.1) | 2.5 | 3.9 | 9.6 (3.3) | 2.5 | 7.1 | 8.1 (3.8) | 2.5 | 5.6 |
| **n-6 fatty acids (g)** | 14 (11) | 0 | 14 | 21 (12) | 0 | 21 | 18 (14) | 0 | 18 |

DACH = Germany, Austria, Switzerland
